# Macropinocytosis is an alternative pathway of cysteine acquisition and mitigates sorafenib-induced ferroptosis in hepatocellular carcinoma

**DOI:** 10.1186/s13046-022-02296-3

**Published:** 2022-03-14

**Authors:** Jun-Kyu Byun, Seunghyeong Lee, Gil Won Kang, Yu Rim Lee, Soo Young Park, Im-Sook Song, Jae Won Yun, Jaebon Lee, Yeon-Kyung Choi, Keun-Gyu Park

**Affiliations:** 1 Research Institute of Aging and Metabolism, Kyungpook National University, Daegu, Korea; 2 Department of Internal Medicine, School of Medicine, Kyungpook National University, Kyungpook National University Hospital, Daegu, Korea; 3 Department of Biomedical Science, Graduate School, Kyungpook National University, Daegu, Korea; 4 BK21 FOUR KNU Convergence Educational Program of Biomedical Sciences for Creative Future Talents, School of Medicine, Kyungpook National University, Daegu, Korea; 5 Research Institute of Pharmaceutical Science, College of Pharmacy, Kyungpook National University, Daegu, Korea; 6 Veterans Medical Research Institute, Veterans Health Service Medical Center, Seoul, Korea; 7 Sungkyunkwan University School of Medicine, Seoul, Korea

**Keywords:** Hepatocellular carcinoma, Sorafenib, Macropinocytosis, Ferroptosis, Sorafenib resistance

## Abstract

**Background:**

Macropinocytosis, an important nutrient-scavenging pathway in certain cancer cells, allows cells to compensate for intracellular amino acid deficiency under nutrient-poor conditions. Ferroptosis caused by cysteine depletion plays a pivotal role in sorafenib responses during hepatocellular carcinoma (HCC) therapy. However, it is not known whether macropinocytosis functions as an alternative pathway to acquire cysteine in sorafenib-treated HCC, and whether it subsequently mitigates sorafenib-induced ferroptosis. This study aimed to investigate whether sorafenib drives macropinocytosis induction, and how macropinocytosis confers ferroptosis resistance on HCC cells.

**Methods:**

Macropinocytosis, both in HCC cells and HCC tissues, was evaluated by measuring TMR-dextran uptake or lysosomal degradation of DQ-BSA, and ferroptosis was evaluated via C11-BODIPY fluorescence and 4-HNE staining. Sorafenib-induced ferroptosis and macropinocytosis were validated in tumor tissues taken from HCC patients who underwent ultrasound-guided needle biopsy.

**Results:**

Sorafenib increased macropinocytosis in human HCC specimens and xenografted HCC tissues. Sorafenib-induced mitochondrial dysfunction was responsible for activation of PI3K-RAC1-PAK1 signaling, and amplified macropinocytosis in HCC. Importantly, macropinocytosis prevented sorafenib-induced ferroptosis by replenishing intracellular cysteine that was depleted by sorafenib treatment; this rendered HCC cells resistant to sorafenib. Finally, inhibition of macropinocytosis by amiloride markedly enhanced the anti-tumor effect of sorafenib, and sensitized resistant tumors to sorafenib.

**Conclusion:**

In summary, sorafenib induced macropinocytosis, which conferred drug resistance by mitigating sorafenib-induced ferroptosis. Thus, targeting macropinocytosis is a promising therapeutic strategy to facilitate ferroptosis-based therapy for HCC.

**Graphic Abstract:**

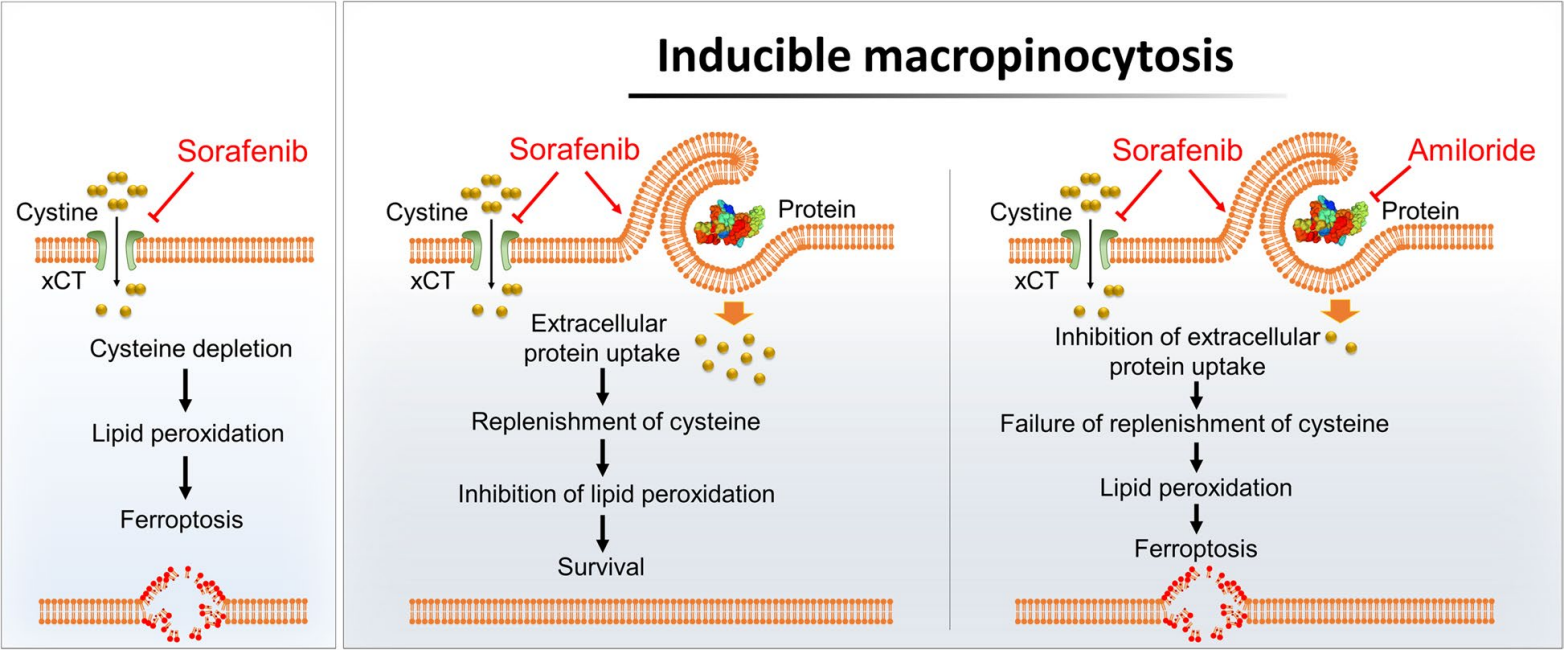

**Supplementary Information:**

The online version contains supplementary material available at 10.1186/s13046-022-02296-3.

## Background

Hepatocellular carcinoma (HCC), one of the most common and fatal malignancies worldwide [1], is usually diagnosed at an advanced stage, meaning that surgical resection or local therapy is not possible [2]. The therapeutic option is sorafenib, a multi-target kinase inhibitor, which has been the standard treatment for advanced HCC [3, 4]. Despite recent advances in immunomodulating therapy for HCC, sorafenib remains the most effective single drug therapy [5]. However, high recurrence rates and acquired resistance result in limited survival benefits [6]. Thus, a novel strategy to improve sorafenib responses in liver cancer is required.

Ferroptosis is an iron-dependent, oxidative form of cell death characterized morphologically by membrane “ballooning” coupled with the presence of relatively intact nuclei [7]. Increasing evidence suggests that dysfunction of ferroptosis is highly related to initiation and development of various diseases, including cancer [8]. Ferroptosis is induced by inhibition of cystine/glutamate antiporter SLC7A11/xCT activity, downregulation of glutathione peroxidase 4, and accumulation of iron and lipid reactive oxygen species (ROS) [9]. Given that intracellular cysteine deficiency via inhibition of xCT activity mediates sorafenib-induced ferroptosis in HCC [10, 11], the existence of alternative pathways for acquisition of cysteine by HCC are potentially responsible for the limited antitumor effects of sorafenib.

Macropinocytosis is a process by which cells non-selectively engulf extracellular fluid and its contents through large uncoated vacuoles called macropinosomes [12]. Internalized extracellular proteins are degraded in a lysosome-dependent fashion; this is an alternative pathway that contributes to the intracellular amino acid pool [13, 14]. Cancer cells carrying oncogenic mutations in KRAS or PTEN induce macropinocytosis, thereby allowing them to survive and proliferate under conditions in which amino acids are limiting [13, 15]. Recent studies show that PI3K and AMPK-activated conditions enable proliferating cells without oncogenic mutations to trigger macropinocytosis, thereby adapting to the changed microenvironment [16, 17]. Sorafenib exerts antitumor effects by targeting multiple kinases (e.g., VEGFR, PDGFR, and RAF kinases) as well as xCT, and by activating PI3K and AMPK pathways implicated in sorafenib resistance [6, 18]; however, it is largely unknown whether sorafenib induces macropinocytosis in HCC and whether macropinocytosis functions as an alternative pathway for acquisition of cysteine to prevent sorafenib-induced ferroptosis and reduce susceptibility to sorafenib.

Here, we investigated whether sorafenib drives macropinocytosis induction, and how macropinocytosis confers resistance to ferroptosis, in HCC. Mechanistically, we identified a critical role for macropinocytosis as a negative modulator of ferroptosis, and demonstrated that combined treatment with a macropinocytosis inhibitor increased the therapeutic effect of sorafenib in HCC. These results suggest that pharmacological blockade of macropinocytosis represents a promising strategy for ferroptosis-based HCC therapy.

## Materials and methods

### Patients and specimens

Samples of human liver cancer tissues were obtained from 11 HCC patients who underwent ultrasound-guided needle biopsy at Kyungpook National University Hospital in 2021. Fresh tissue specimens were placed in DMEM and dissected as quickly as possible prior to performing the macropinocytosis assay. Patients did not receive preoperative anticancer treatments such as treatment with sorafenib, transarterial chemoembolization, or local ablation therapy. The study was approved by the institutional review board (IRB) of Kyungpook National University Hospital (IRB no. KNUH 2014–04-056–015).

### Ex vivo macropinocytosis assay

To detect and quantify macropinocytosis in tumor tissues, ex vivo labeling of macropinosomes in tumor tissues was performed. Human liver cancer tissues obtained from surgical resections were dissected into approximately 3 mm lengths and immersed in DMEM containing 10% FBS and 1% P/S at 37 °C/5% CO_2_. Next, after 24 h of treatment with 10 μM sorafenib, tissues were exposed to TMR-dextran 70 kDa (0.5 mg/ml; Thermo Fisher Scientific, Waltham, MA, USA) for 1 h. Prior to detection of macropinocytosis in tumor xenograft tissues (Huh7 and SK-Hep1) isolated from mice, animals received daily intraperitoneal (i.p.) injections of vehicle containing amiloride (5 mg/kg) and/or sorafenib (5 mg/kg) for 3 days; the injections were given when the volume of Huh7 and SK-Hep1 xenograft tumors reached about 300 mm^3^. Tumor tissues were harvested and cut into slices. Tissues were immersed in 1 mg/ml of TMR-dextran 70 kDa for 1 h, washed three times in phosphate-buffered saline (PBS), and frozen in optimal cutting temperature compound. The frozen tumor tissues were processed and macropinocytosis was quantified as described previously [19]. Orthotopic liver tumor tissues from RIL-175 mice were harvested at the experimental endpoint, sliced, and processed as described above.

### In vitro macropinocytosis assay

Cancer cells were treated with TMR-dextran 70 kDa (0.5 mg/mL) for 1 h or with DQ-BSA (0.5 mg/mL; Thermo Fisher Scientific) for 4 h. Cancer cells were washed three times with PBS and then fixed with 4% paraformaldehyde. After fixation, cells were mounted in mounting solution containing DAPI (Vector Laboratories, Burlingame, CA, USA). Macropinocytosis was quantified as described previously [19]. To inhibit TMR-dextran uptake or lysosomal degradation of DQ-BSA, cells were pretreated by exposure to a low temperature (4 °C) or to NH_4_Cl (Sigma) for 15 min before addition of chemicals. The number of labeled macropinosomes was analyzed using the ‘Analyze Particle’ tool in Image J (a Java-based image processing program), and fluorescence intensity was determined by measuring the integrated signal density/cell in DQ-BSA-treated cells.

### Immunofluorescence analysis

HCC cell lines, frozen sections of human tumor tissues, and xenograft tumors on glass slides were fixed for 15 min in 4% paraformaldehyde and then permeabilized with 0.3% Triton x-100 for 10 min at room temperature. Following 1 h of blocking in 5% normal goat serum (Vector Laboratories, Burlingame, USA), tissues were incubated overnight at 4 °C with a primary anti-Hepatocyte Specific Antigen antibody, anti-4-HNE (Abcam, 1:100), anti-phospho-AKT, anti-phospho-PAK1, or anti-cleaved caspase-3 (Cell Signaling Technology, 1:200) antibodies. After washing, the tissues were incubated for 1 h at room temperature with Alexa Fluor™ 488 goat-anti-rabbit or 568 goat-anti-rabbit (Thermo Fisher Scientific) secondary antibodies. Nuclei were staining with DAPI (Vector Laboratories). Fluorescence intensity was determined by obtaining the integrated signal density/cell in each tumor tissue.

## Results

### Sorafenib induces macropinocytosis in human HCC tissues and HCC cells

**Fig. 1:**
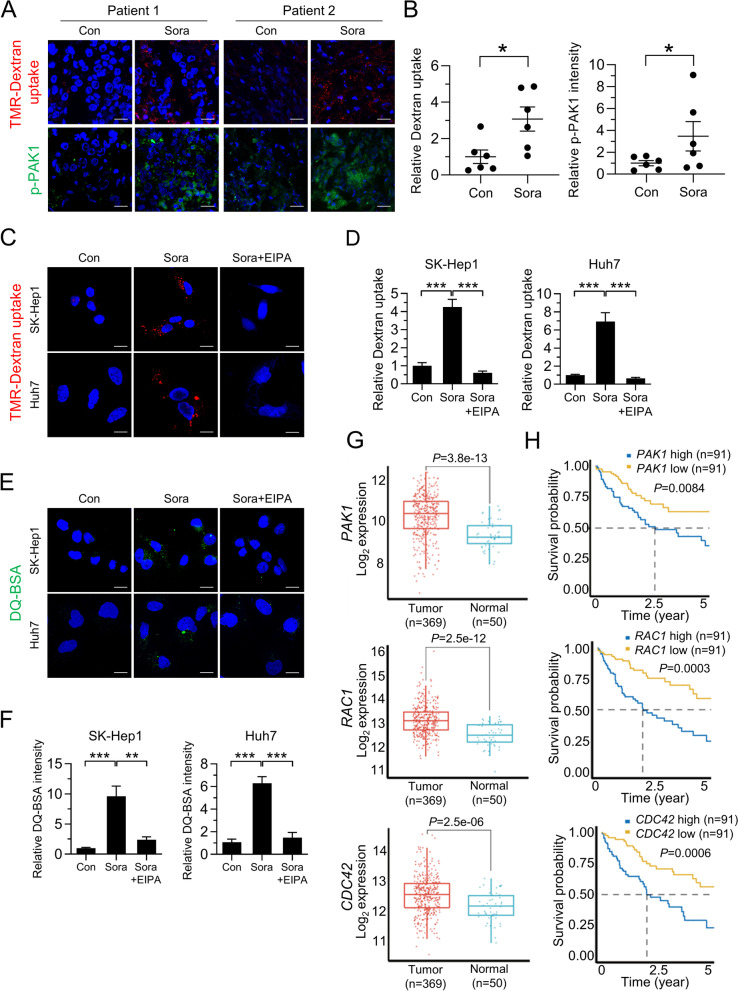
Sorafenib induces macropinocytosis in human HCC tissues and HCC cells. **A** Images of macropinocytotic uptake of labeled TMR-dextran (red), immunofluorescence staining with anti-phosphorylated PAK1 (green), and **B** quantification of macropinosomes and phosphorylated PAK1 fluorescence in response to sorafenib in tumor tissues from six treatment-naïve HCC patients. **C** Representative images of macropinosomes (red) in sorafenib-treated liver cancer cells in the presence or absence of EIPA, an inhibitor of macropinocytosis, and **D** quantification of macropinosomes in the cells shown in Fig. 1C. **E** Representative images of DQ-BSA fluorescence (green) in sorafenib-treated liver cancer cells in the presence or absence of EIPA, and **F** quantification of DQ-BSA fluorescence in the cells shown in Fig. 1E. **G** TCGA analysis of *PAK1*, *RAC1*, and *CDC42* expression in human HCC tumor (*n* = 369) and non-tumor tissues (*n* = 50). **H** Correlation between *PAK1*, *RAC1*, or *CDC42* expression and overall survival of human HCC patients (*n* = 91). Data are normalized against values measured in vehicle-treated cells (Con) and expressed as the mean ± SEM of at least three independent experiments. Scale bar, 20 µm. *p < 0.05; **p < 0.01, ***p < 0.001

To determine whether sorafenib induces macropinocytosis in HCC, we assessed alterations in macropinocytic uptake in tumor tissues obtained from treatment-naïve HCC patients. Clinical diagnostics were performed, including immunofluorescence staining for the hepatocellular marker HepPar-1 (Table S1 and Fig. S1A). Macropinosomes were detected using tetramethylrhodamine-labeled high-molecular-mass dextran (TMR-dextran), an established marker of macropinocytosis [19]. Notably, TMR-dextran uptake by human HCC tissues treated with sorafenib was significantly higher than by tissues treated with vehicle (Fig. 1A, B and Fig. S1B). Consistent with a role for PAK1 in regulating inducible macropinocytosis [20], we observed a marked increase in phosphorylated PAK1 in sorafenib-treated human HCC tissues (Fig. 1A, B and Fig. S1B). Sorafenib-induced robust macropinocytic TMR-dextran uptake was further confirmed in multiple HCC cell lines including SK-Hep1, Huh7, PLC/PRF/5, and Hep3B cells; this was attenuated significantly by treatment with a pharmacological blocker of macropinocytosis, 5-(N-ethyl-N-isopropyl)-amiloride (EIPA), which inhibits plasma membrane Na^+^/H^+^ exchangers (NHEs) (Fig. 1C, D and S1C). Increased uptake of macropinocytic TMR-dextran induced by sorafenib was also observed in renal cancer cells Caki-1 and ACHN, suggesting that sorafenib-induced macropinocytosis occurs in other cancer cells (Fig. S1C). To determine whether internalized albumin is degraded intracellularly, we used boron-dipyrromethene (BODIPY)-conjugated BSA (DQ-BSA), which is taken up by macropinocytosis and fluoresces after lysosomal degradation [21]. Indeed, sorafenib-treated HCC and renal cancer cells showed increased DQ-BSA fluorescence, which was attenuated by EIPA (Fig. 1E, F and Fig. S1D). Furthermore, sorafenib-induced dextran or DQ-BSA uptake was attenuated at 4 °C, or by treatment with NH_4_Cl (an uncoupler of lysosomal acidification), respectively, which also supports the notion that sorafenib-induced uptake and degradation of extracellular proteins is mediated by macropinocytosis (Fig. S1E and F).

Consistent with the role of RAC1, PAK1, and CDC42 in regulating inducible macropinocytosis [22], analysis of the TCGA dataset revealed higher expression of RAC1, PAK1, and CDC42 in HCC tissues than in non-tumor lesions (Fig. 1G). High levels of RAC1, PAK1, and CDC42 expression correlated with shorter overall survival (Fig. 1H). When these clinical results are considered alongside induction of macropinocytosis by sorafenib, it appears that levels of macropinocytosis in HCC predict prognosis and, furthermore, that induction of macropinocytosis may be associated with the outcome of sorafenib treatment.

### Macropinocytosis-mediated acquisition of cysteine enables HCC cells to acquire resistance to sorafenib-induced ferroptosis

**Fig. 2:**
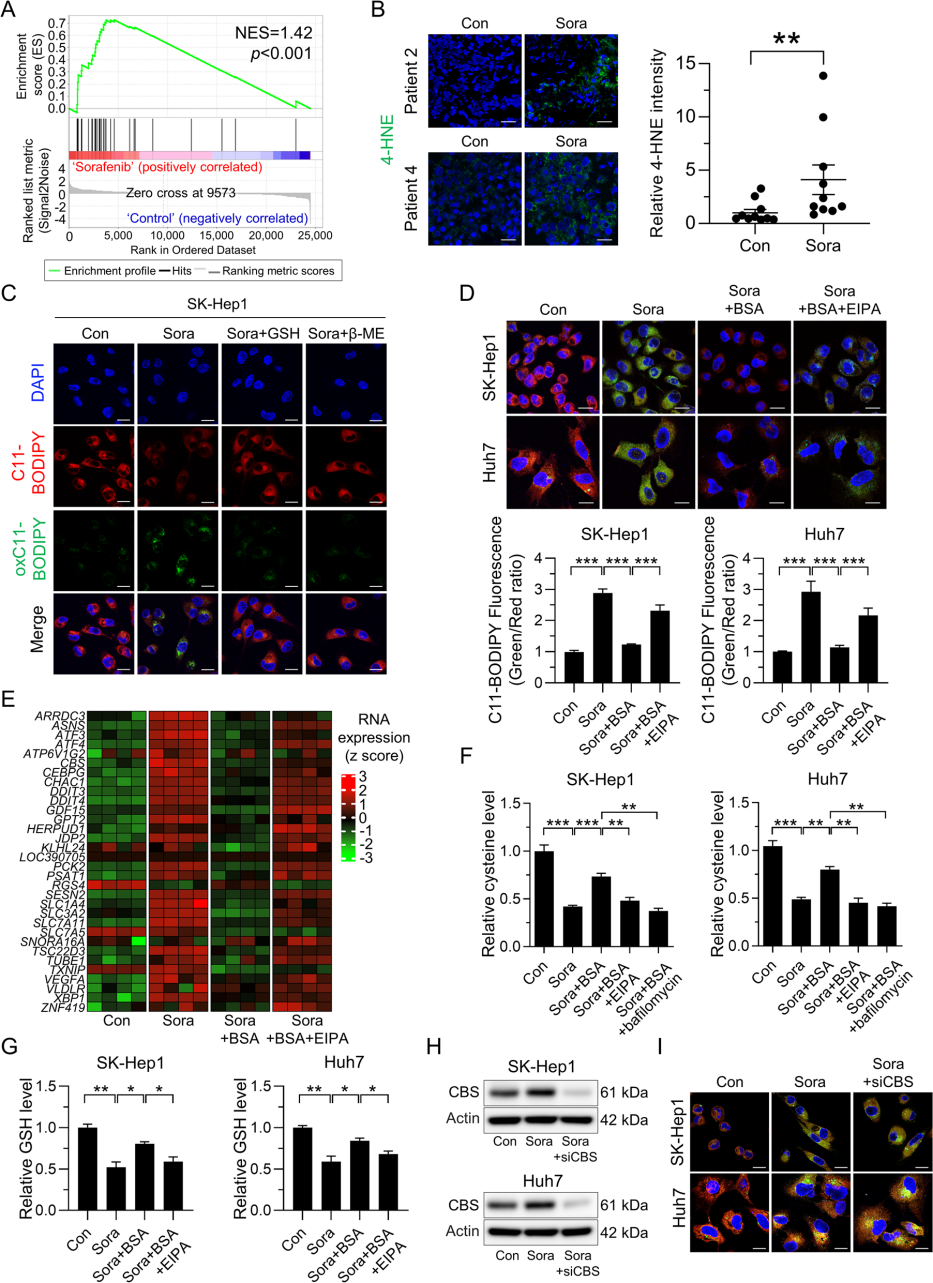
Macropinocytosis-mediated acquisition of cysteine rescues sorafenib-induced ferroptosis. **A** Gene set enrichment analysis of published ferroptosis expression signatures among genes expressed by sorafenib-treated SK-Hep1 cells. **B** Representative immunofluorescence staining with anti-4-HNE (left panel) and quantification of 4-HNE fluorescence in response to sorafenib in tumor tissues from ten treatment-naïve HCC patients (right panel). **C** Representative images of C11-BODIPY, a marker of lipid peroxidation, in SK-Hep1 cells treated with sorafenib, either alone or in combination with the antioxidant GSH or β-mercaptoethanol (β-ME). C11-BOIPY represents levels of staining with the probe (unoxidized), while oxC11-BODIPY (oxidized) indicates the levels of lipid ROS. **D** Representative images of C11-BODIPY in SK-Hep1 and Huh7 cells treated with sorafenib, either alone or in combination with BSA and/or EIPA (upper panel). Quantification of C11-BODIPY fluorescence in cells (lower panel). **E** Genes shown in the heatmap of SK-Hep1 cells treated with sorafenib, either alone or in combination with BSA or/and EIPA. **F** Relative cysteine levels in SK-Hep1 and Huh7 cells treated with sorafenib, either alone or in combination with BSA, EIPA, and/or bafilomycin A1, an inhibitor of protein degradation in lysosomes. **G** Relative GSH levels in SK-Hep1 and Huh7 cells treated with sorafenib, either alone or in combination with BSA and/or EIPA. **H** Effect of sorafenib on CBS proteins in control siRNA or siCBS-transfected SK-Hep1 and Huh7 cells. **I** Representative images of C11-BODIPY in SK-Hep1 and Huh7 cells shown in Fig. 2H. Data are normalized against values measured in vehicle-treated cells (Con) and expressed as the mean ± SEM of at least three independent experiments. Scale bar, 20 µm. *p < 0.05, **p < 0.01, and *** *p* < 0.001

The findings described above prompted further investigations to determine the effect of macropinocytosis induction on sorafenib-induced ferroptosis. Consistent with previous results [10, 11], we found that genes up-regulated in response to sorafenib were enriched in a ferroptosis-related gene signature from human fibrosarcoma cells treated with the selective xCT inhibitor erastin (Fig. 2A) [23]. Analysis of tumor tissues from HCC patients revealed increased staining of 4-HNE, a byproduct of lipid peroxidation, after sorafenib treatment (Fig. 2B and Fig. S2A). Given that sorafenib inhibits SLC7A11 directly and consequently abolishes glutathione (GSH) biosynthesis and induces ferroptosis [24], we monitored lipid ROS accumulation, which is a hallmark of ferroptosis, using C11-BODIPY. As expected, sorafenib increased lipid ROS accumulation in HCC cells, whereas supplementation with GSH, or β-mercaptoethanol (β-ME) which promotes cystine uptake in an xCT-independent manner [25], prevented sorafenib-induced lipid ROS accumulation (Fig. 2C and Fig.S2B). Because lysosomal catabolism of extracellular albumin taken up by macropinocytosis plays a role in maintaining intracellular amino acid availability, we reasoned that uptake of extracellular albumin might prevent ferroptosis in the presence of sorafenib. To test this idea, we assessed lipid ROS accumulation in sorafenib-treated HCC cells with and without albumin supplementation. We found that addition of BSA reduced sorafenib-induced ferroptosis, as evidenced by attenuated lipid oxidation and 4-HNE staining intensity, whereas the inhibitory effect of BSA on sorafenib-induced ferroptosis was suppressed by EIPA (Fig. 2D and Fig. S2C). EIPA alone did not increase accumulation of lipid ROS, suggesting that EIPA increased ferroptosis by inhibiting sorafenib-induced macropinocytosis (Fig. S2D and E). In line with this finding, mRNA sequencing data revealed no upregulation of ferroptosis-related genes in sorafenib-treated HCC cells in the presence of BSA; BSA had no effect when macropinocytosis was inhibited by EIPA (Fig. 2E). Consistent with previous results showing that sorafenib induced ferroptosis by depletion of cellular cysteine, treatment with sorafenib decreased intracellular cysteine levels in HCC cells (Fig. 2F). However, in the presence of BSA, HCC cells recovered from sorafenib-induced cysteine depletion significantly, which was reversed by treatment with EIPA or bafilomycin (Fig. 2F). We also observed that intracellular GSH levels correlated with cysteine levels in the presence or absence of BSA and a macropinocytosis inhibitor (Fig. 2G). Given that cysteine is used to synthesize GSH required to mitigate ferroptosis, we examined whether inhibiting GSH synthesis or lysosomal degradation of BSA abolishes the protective effect of macropinocytosis on sorafenib-induced ferroptosis. Indeed, sorafenib-induced ferroptosis occurred upon treatment with the GSH synthesis inhibitors buthionine sulfoximine (BSO) or bafilomycin in the presence of BSA (Fig. S2F), indicating that macropinocytosis plays a role in preserving intracellular cysteine levels and preventing ferroptosis in sorafenib-treated HCC cells. Given the contribution of the transsulfuration pathway to intracellular cysteine levels, we further analyzed expression of cystathionine-β-synthase (CBS) and cystathionine γ-lyases (CTH), two enzymes involved in the transsulfuration pathway. We also examined expression of genes involved in the methionine cycle in sorafenib-treated HCC cells. Expression of CBS and CTH increased in response to sorafenib, whereas that of genes involved in methionine cycle were regulated inconsistently (Fig. S2G), which is discrepant with our finding that sorafenib depletes intracellular cysteine. Furthermore, in contrast to depletion of intracellular cysteine by EIPA in the presence of BSA, RNA-seq analyses revealed that expression of CBS and CTH in response to EIPA tended to remain high (Fig. S2H). Notably, although CBS was upregulated by sorafenib, it did not prevent lipid ROS accumulation, nor did silencing of CBS amplify sorafenib-induced lipid ROS accumulation significantly (Fig. 2H, I and Fig. S2I). Taken together, these data reveal that the transsulfuration pathway cannot compensate for the sorafenib-induced deficit of intracellular cysteine, and that macropinocytosis plays a pivotal role in preserving intracellular cysteine and GSH levels, which support the fitness of HCC cells and allows them to escape the deleterious effects of ferroptosis.

### Sorafenib-induced mitochondrial dysfunction drives macropinocytosis by activating PI3K-RAC1-PAK1 signaling

**Fig. 3:**
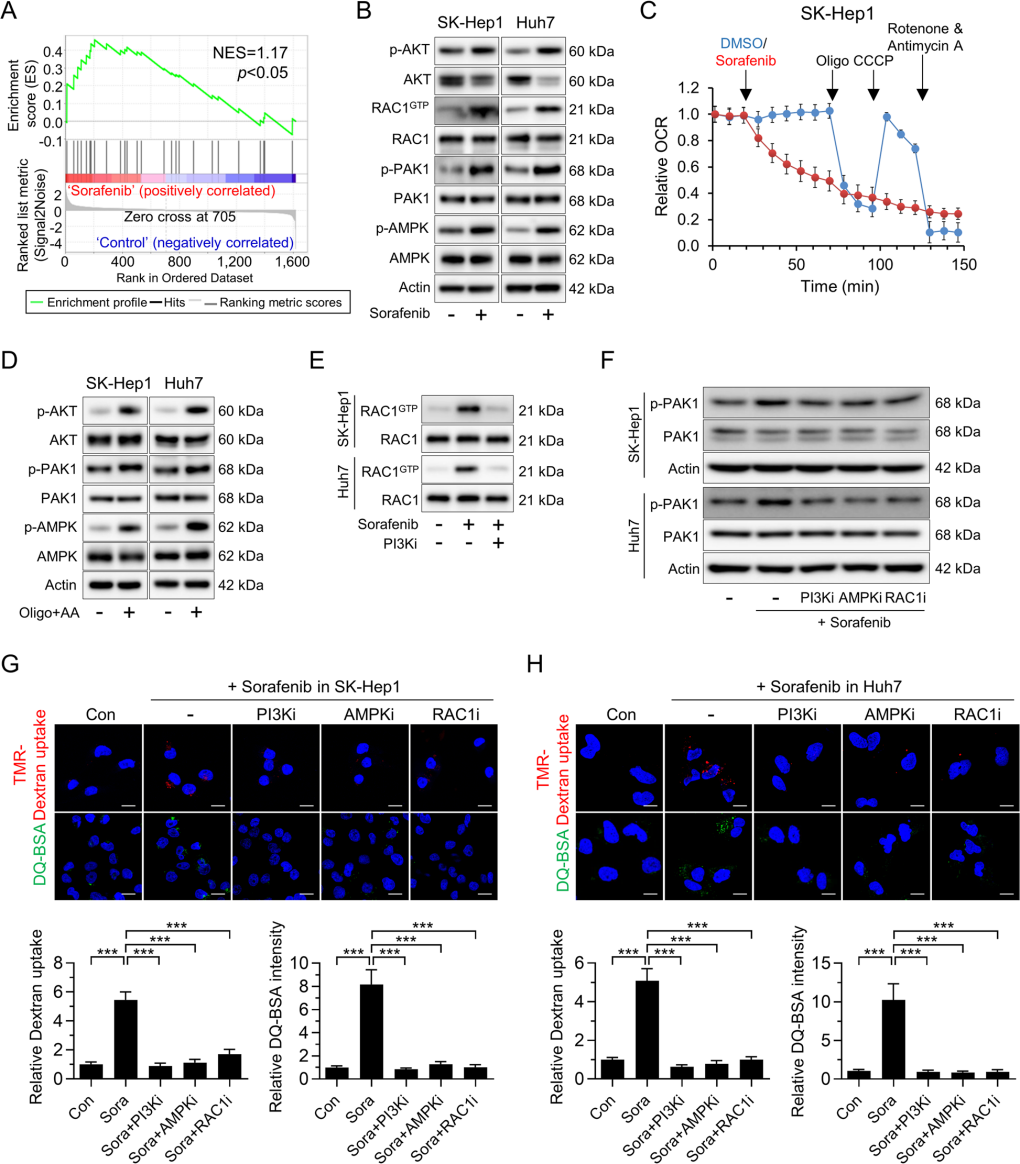
Sorafenib induces macropinocytosis via mitochondrial dysfunction-induced activation of PI3K-RAC1-PAK1 signaling. **A** Gene set enrichment analysis of PI3K signaling related genes expressed by sorafenib-treated SK-Hep1 cells. **B** Levels of phosphorylated AKT, activation of RAC1, and phosphorylated PAK1 and AMPK, in SK-Hep1 and Huh7 treated with sorafenib. **C** The oxygen consumption rate (OCR) of SK-Hep1 at the indicated time points after sorafenib treatment. **D** Levels of phosphorylated AKT, PAK1, and AMPK in SK-Hep1 and Huh7 cells after treatment with oligomycin (Oligo) and antimycin A (AA). **E** Effect of a PI3K inhibitor (LY294002) on RAC1 activation in sorafenib-treated SK-Hep1 and Huh7 cells. **F** Levels of phosphorylated PAK1 in sorafenib-treated SK-Hep1 and Huh7 cells in the presence or absence of a PI3K inhibitor (LY294002), an AMPK inhibitor (compound c), or a RAC1 inhibitor (NSC23766). (**G** and **H**) Representative images of macropinosomes (red), and DQ-BSA fluorescence (green) in sorafenib-treated SK-Hep1 (**G**) and Huh7 (**H**) in the presence or absence of a PI3K inhibitor, an AMPK inhibitor, or a RAC1 inhibitor (upper panel). Quantification of macropinosomes and DQ-BSA fluorescence in cells (lower panel). Data are normalized against values measured in vehicle-treated cells (Con) and expressed as the mean ± SEM of at least three independent experiments. Scale bar, 20 µm. ***p < 0.001

To investigate the mechanisms by which sorafenib drives macropinocytosis in HCC cells, we investigated whether key regulators of macropinocytosis (such as PI3K, RAS, and WNT) increased in HCC cells exposed to sorafenib [3]. Notably, RNA-seq profiling of sorafenib-treated HCC cells revealed that genes activated by PI3K were highly enriched (Fig. 3A). By contrast, RAS and WNT signaling was not altered significantly upon sorafenib treatment (Fig. S3A). PI3K affects RAC1 activity by regulating the activity of PIP3-dependent RAC1 guanine nucleotide exchange factors, and activated RAC1 binds to and stimulates the kinase activity of PAK1 via intramolecular inhibition and consequent autophosphorylation, which drives macropinocytosis through instigation of macropinocytic induction mechanisms [26–28]. Indeed, we found that sorafenib-treated cells increased PI3K activity by increasing expression of the phosphorylated PIP3-dependent Ser/Thr kinase AKT and found it increased levels of RAC1-GTP and phosphorylation of PAK1 (Fig. 3B).

To further elucidate the mechanism responsible for sorafenib-induced macropinocytosis, we firstly examined whether xCT inhibition is implicated in macropinocytosis of HCC cells. As shown in Fig. S3B, erastin did not increase dextran uptake in HCC cells, suggesting xCT-independent macropinocytosis by sorafenib. Considering that sorafenib targets mitochondrial biogenesis, we next examined the impact of sorafenib-induced mitochondrial dysfunction on macropinocytosis in HCC cells. As expected, sorafenib decreased mitochondrial oxygen consumption by HCC cells (Fig. 3C and Fig. S3C). Since sorafenib induces mitochondrial dysfunction by inhibiting complex II/III in the electron transporter chain, as well as ATP synthase [29], we investigated whether co-treatment with oligomycin and antimycin increases expression of major kinases required for macropinocytosis induction in HCC cells. In parallel with previous findings, we observed that HCC cells treated with oligomycin and antimycin exhibited increased phosphorylation of AKT and PAK1, accompanied by increased TMR-dextran uptake and DQ-BSA fluorescent puncta (Fig. 3D and Fig. S3D, E). After demonstrating that mitochondrial dysfunction is implicated in macropinocytosis induction in sorafenib-treated HCC cells, we investigated the effects of PI3K inhibition on sorafenib-induced macropinocytosis. The results showed that the sorafenib-induced increase in RAC1-GTP levels was strongly suppressed by treatment with a PI3K inhibitor, suggesting PI3K-dependent RAC1 regulation in HCC cells (Fig. 3E). Furthermore, sorafenib increased PAK1 phosphorylation and TMR-dextran or DQ-BSA uptake, which were strongly suppressed by treatment with PI3K or RAC1 inhibitors (Fig. 3F, G and H). Finally, sorafenib-induced macropinocytosis in HCC cells was abrogated by a PAK1 inhibitor (Fig. S3F). Given that sorafenib activates AMPK indirectly by inhibiting mitochondrial function [30], and the requirement for AMPK to support RAC1 activation and macropinosome formation [15, 17], we also investigated the roles of AMPK in sorafenib-induced macropinocytosis. Treatment with sorafenib, or co-treatment with oligomycin and antimycin, increased phosphorylation of AMPK, while an AMPK inhibitor abrogated both sorafenib-induced RAC1 activity (as measured by inhibition of PAK1 phosphorylation) and induction of macropinocytosis (Fig. 3B, D, F, G and H).

### Inhibiting macropinocytosis with amiloride recovers sorafenib-induced ferroptosis in HCC

**Fig. 4:**
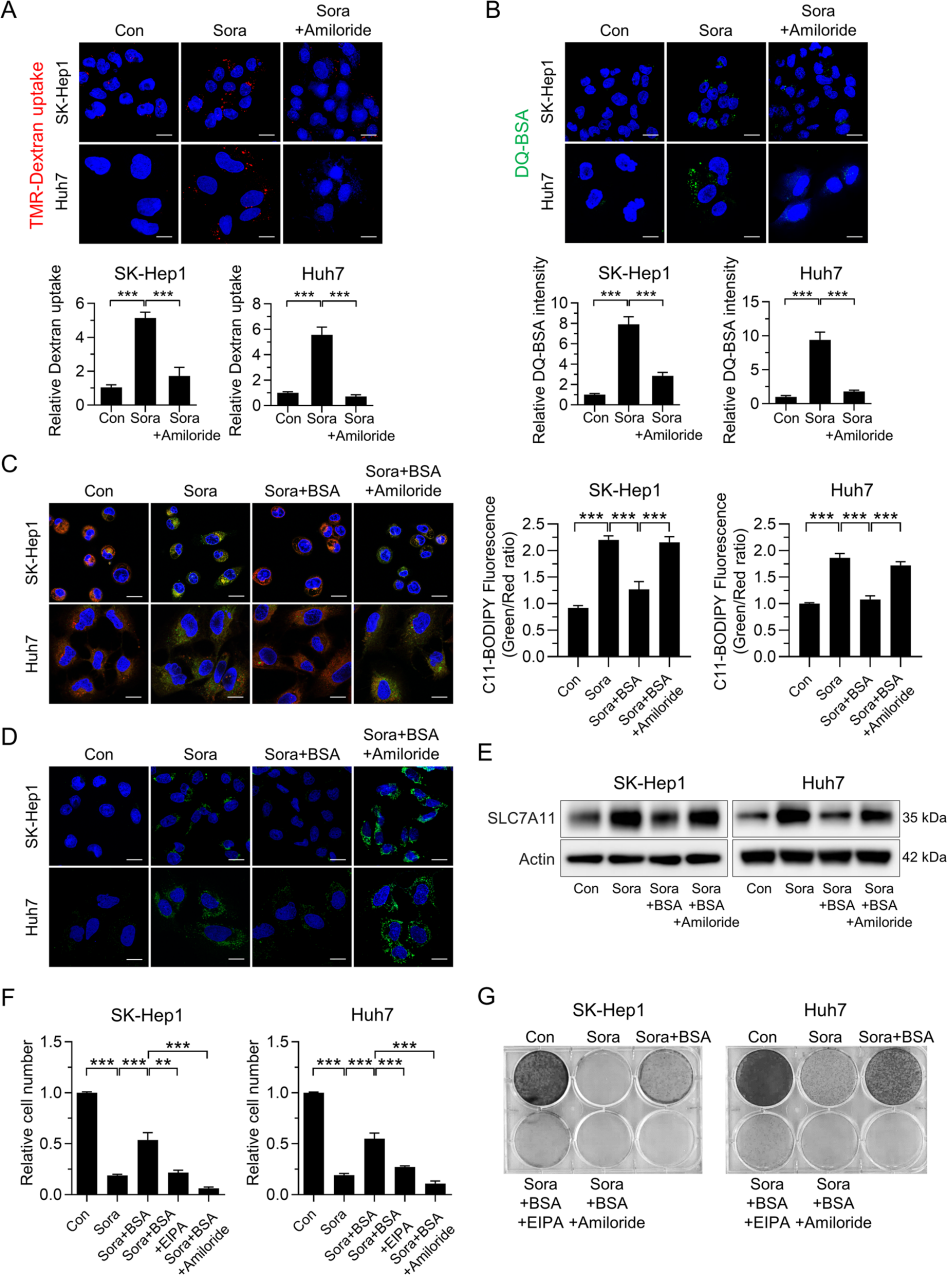
Inhibition of macropinocytosis increases sorafenib-induced ferroptosis in HCC. **A** Representative images of macropinosomes (red) in sorafenib-treated SK-Hep1 and Huh7 cells in the presence or absence of amiloride (upper panel). Quantification of macropinosomes in cells (lower panel). **B** Representative images of DQ-BSA fluorescence (green) in sorafenib-treated SK-Hep1 and Huh7 cells in the presence or absence of amiloride (upper panel). Quantification of DQ-BSA fluorescence in cells (lower panel). **C** Representative images of C11-BODIPY in SK-Hep1 and Huh7 cells treated with sorafenib, either alone or in combination with BSA and/or amiloride (left panel). Quantification of C11-BODIPY fluorescence in cells (right panel). **D** Immunofluorescence of 4-HNE staining in SK-Hep1 and Huh7 cells treated with sorafenib, either alone or in combination with BSA and/or amiloride (green). **E** Level of SLC7A11 in SK-Hep1 and Huh7 cells treated with sorafenib, either alone or in combination with BSA and/or amiloride. **F** Relative number of SK-Hep1 and Huh7 cells treated for 48 h with sorafenib, either alone or in combination with BSA, EIPA, and/or amiloride. **G** Clonogenic assay of SK-Hep1 and Huh7 cells treated for 7 days with sorafenib, either alone or in combination with BSA, EIPA, and/or amiloride. Data are normalized against values measured in vehicle-treated cells (Con) and expressed as the mean ± SEM of at least three independent experiments. Scale bar, 20 µm. **p < 0.01, and ***p < 0.001

After demonstrating that macropinocytosis protects sorafenib-treated HCC cells from ferroptosis, we investigated whether blocking macropinocytosis with amiloride, a clinically feasible NHE inhibitor, potentiates the antitumor effect of sorafenib [31]. Indeed, sorafenib-induced macropinocytosis was attenuated by treatment with amiloride (Fig. 4A and B). Furthermore, blocking macropinocytosis with amiloride potentiated ferroptosis, as evidenced by increased lipid oxidation and 4-HNE staining intensity; these were prevented by cotreatment with ferroptosis inhibitors ferrostatin-1 (a lipophilic antioxidant) or deferoxamine (an iron chelator) (Fig. 4C, D and Fig. S4A, B). Previous results show compensatory transcriptional upregulation of SLC7A11 upon inhibition of system xc − [7, 32]; thus, we next examined alteration of SLC7A11 expression in response to amiloride in the presence of sorafenib. Consistent with previous studies, we found substantial upregulation of SLC7A11 in sorafenib-treated HCC cells (Fig. 4E). Supplementation with BSA reduced sorafenib-induced upregulation of SLC7A11, which was reversed when sorafenib was combined with amiloride (Fig. 4E). These findings, combined with results shown in Fig. 4C and D, suggested that transcriptional upregulation of SLC7A11 is not sufficient to prevent ferroptosis in sorafenib-treated HCC cells. Next, we investigated the contribution of ferroptosis in sorafenib-induced downregulation of HCC cell numbers. As shown in Fig. S4C, neither apoptosis nor necroptosis signaling was activated in sorafenib-treated HCC cells, consistent with previous findings [24]. Rather, sorafenib-induced decreases in HCC cell numbers were reversed significantly by co-treatment with ferroptosis inhibitors ferrostatin-1, trolox, and GSH, but not by treatment with the apoptosis inhibitor Z-VAD-FMK or the necroptosis inhibitor necrostatin-1 (Fig. S4D and E). In accordance with these results, recovery of HCC cell numbers in the presence of BSA was abolished when macropinocytosis was inhibited by amiloride or EIPA, which do not affect apoptosis or necroptosis signaling significantly (Fig. 4F and Fig. S4F, G). A clonogenic assay also confirmed that macropinocytosis renders HCC cells resistant to sorafenib treatment (Fig. 4G).

### Inhibiting macropinocytosis with amiloride increases the antitumor efficacy of sorafenib

**Fig. 5:**
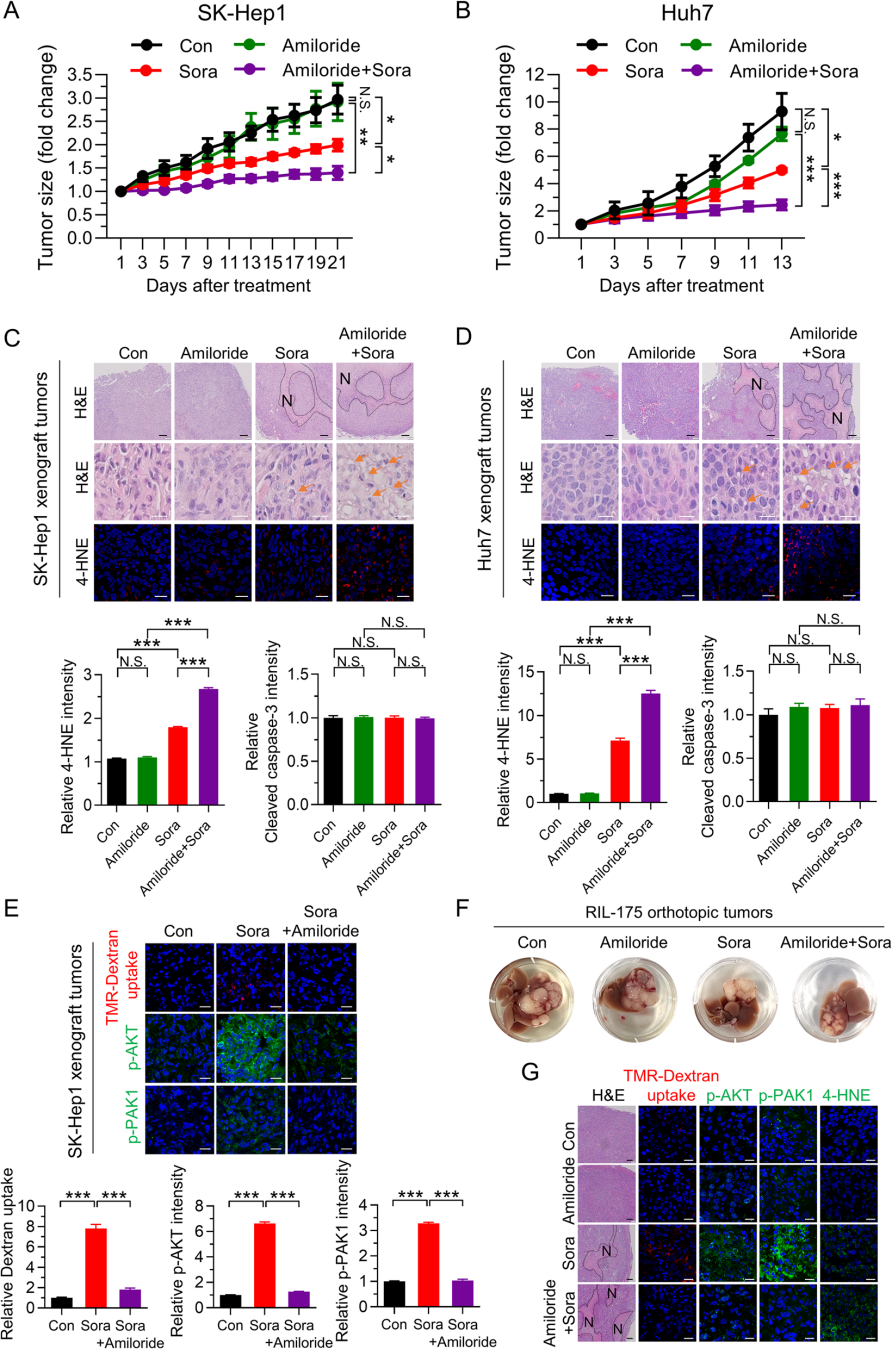
Inhibition of macropinocytosis by amiloride augments sorafenib-induced inhibition of tumor growth. (**A** and **B**) Tumor growth curves. (**C** and **D**) Hematoxylin and eosin (H&E) staining and immunofluorescence staining with anti-4-HNE (scale bar, 20 µm) (upper panel), and quantification of immunofluorescence tissue staining with anti-4-HNE and cleaved caspase-3 (See Fig. S5**E** and **F**) (lower panel) (*n* = 5 per group). Hashed lines and arrows indicate regional necrosis (N) and lipid droplet formation, respectively. (**E**) Representative images of macropinosomes and immunofluorescence staining for anti-phosphorylated AKT and PAK1 in sections of SK-Hep1 xenografted tumor tissue (upper panel). Quantification of macropinosomes, phosphorylated AKT and PAK1 fluorescence in tumor tissues (lower panel) (*n* = 3 per group). (**F**) Representative gross images of orthotopic RIL-175 tumor tissues from 57BL/6 mice after drug treatments (*n* = 6–8 per group). **G** H&E staining (scale bar, 150 µm), representative images of macropinosomes, phosphorylated AKT and PAK1, and 4-HNE staining (scale bar, 20 µm) in sections of RIL-175-orthotophic tumor tissues. Hashed lines indicate regional necrosis (N). Data are normalized against values measured in vehicle-treated tumors (Con) and expressed as the mean ± SEM. Black scale bar, 150 µm; White scale bar, 20 µm. N.S., not significant; *p < 0.05, ** *p* < 0.01, and *** *p* < 0.001

The findings described above suggest that macropinocytosis makes HCC cells resistant to sorafenib-induced ferroptosis; therefore, we investigated whether inhibiting macropinocytosis increases the antitumor activity of sorafenib in vivo. Although treatment of SK-Hep1- or Huh7-bearing mice with sorafenib led to a marked reduction in tumor mass, it did not completely inhibit tumor growth (Fig. 5A, B and Fig. S5A–D). However, simultaneous treatment with sorafenib and amiloride suppressed tumor growth almost completely, without any differences in body weight between the groups (Fig. 5A, B and Fig. S5A–D). In parallel with tumor growth, morphological analysis revealed that combined treatment with sorafenib and amiloride markedly increased the number of necrotic lesions with an unusual cellular phenotype, which was characterized by ballooned cells showing cytoplasmic vacuolization, compared with sorafenib treatment alone (Fig. 5C, D). Further analyses of xenograft tumor tissues by immunofluorescence revealed that sorafenib alone increased 4-HNE staining, but not cleaved caspase-3 staining (Fig. 5C, D and Fig. S5E, F). Importantly, combined treatment with sorafenib and amiloride increased 4-HNE staining significantly without altering cleaved caspase-3 staining in tumor tissues from SK-Hep1- and Huh7-bearing mice (Fig. 5C and D and Fig. S5E, F), indicating that ferroptosis is responsible for the increased therapeutic effect of sorafenib. We further investigated the relevance of macropinocytosis to reduced tumor growth induced by co-treatment with sorafenib and amiloride. Consistent with the in vitro results, we found that combined treatment with amiloride attenuated sorafenib-induced dextran uptake and staining of phosphorylated AKT and PAK1 in HCC tissues from SK-Hep1 or Huh7 xenograft tumors (Fig. 5E and Fig. S5G), which highlights the important role of inhibiting macropinocytosis in increasing the susceptibility of HCC to sorafenib. The combined effects of sorafenib and amiloride on tumor growth, the area of necrosis, and the levels of phosphorylated AKT, PAK1, and 4-HNE after blocking macropinocytosis were further verified in orthotopic RIL-175 tumors transplanted into C57BL/6 mice (Fig. 5F, G and Fig. S5H, I and J).

### Sorafenib-resistant HCC cells exhibit high levels of macropinocytosis, and inhibiting macropinocytosis sensitizes resistant tumors to sorafenib

**Fig. 6:**
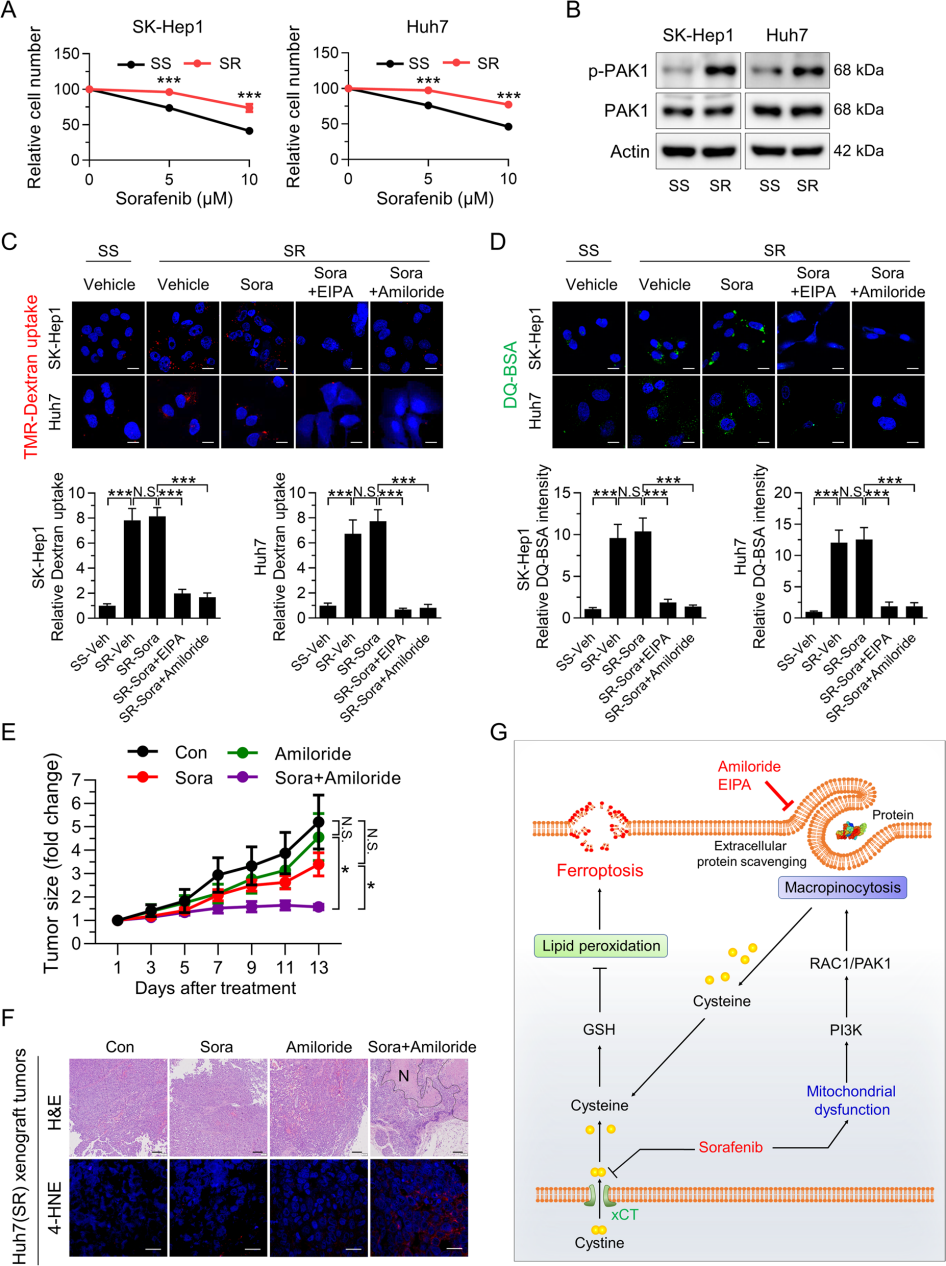
Sorafenib-resistant HCC cells show enhanced macropinocytosis, and amiloride sensitizes resistant HCC tumors to sorafenib. **A** Relative numbers of Huh7 and SK-Hep1 (sorafenib-sensitive, SS) and sorafenib-resistant Huh7 and SK-Hep1 (sorafenib-resistant, SR) cells treated with sorafenib for 24 h. **B** Levels of phosphorylated PAK1 in Huh7 (SS and SR) and SK-Hep1 (SS and SR) cells. **C** and **D** Representative images of macropinosomes (red; **C**) and DQ-BSA fluorescence (green; **D**) in sorafenib-resistant Huh7 (SR) and SK-Hep1 (SR) in the presence or absence of EIPA or amiloride (scale bar, 20 µm). **E** Growth curve of Huh7 (SR)-xenografted tumors after drug treatment. **F** H&E staining (scale bar, 150 µm) and immunofluorescence staining of tumor tissues with anti-4-HNE (scale bar, 20 µm). Hashed lines indicate regional necrosis (N). (**G**) Schematic showing the role of macropinocytosis in sorafenib resistance: macropinocytosis mitigates sorafenib-induced ferroptosis in HCC. Data are normalized against values measured in vehicle-treated cells (SS-Veh) or tumors (Con), and expressed as the mean ± SEM of at least three independent experiments, or from independent xenografts (*n* = 3–4 per group). N.S., not significant; *p < 0.05 and *** *p* < 0.001

The role of macropinocytosis in triggering resistance to sorafenib-induced ferroptosis provided us with a rationale to use macropinocytosis inhibition as a method of resensitizing sorafenib-resistant tumors. To this end, sorafenib-resistant SK-Hep1 and Huh7 cells were established via exposure to gradually increasing concentrations of sorafenib (up to 10 μM) for ~ 5 months (Fig. 6A). Interestingly, levels of phosphorylated PAK1 were higher in sorafenib-resistant SK-Hep1 and Huh7 cells than in parental cells (Fig. 6B). Moreover, sorafenib-resistant HCC cells exhibited higher dextran uptake and more DQ-BSA fluorescent puncta than parental cells in the presence or absence of sorafenib; these phenomena were abolished by co-treatment with EIPA or amiloride (Fig. 6C and D), suggesting the potential role of macropinocytosis in acquired sorafenib resistance. Finally, we investigated in vivo co-operativity between amiloride and sorafenib in mice bearing sorafenib-resistant HCC cells. Sorafenib or amiloride alone did not reduce tumor growth, whereas combined treatment with sorafenib and amiloride led to a marked reduction in growth of sorafenib-resistant tumors, with no differences in body weight between groups (Fig. 6E and Fig. S6A and B). Consistent with this, sorafenib alone did not induce necrotic lesions in sorafenib-resistant tumors, whereas it markedly increased the number of necrotic lesions when combined with amiloride (Fig. 6F and Fig. S6C). Increased 4-HNE staining further confirmed that amiloride enhanced the therapeutic effects of sorafenib against HCC by increasing ferroptotic cell death (Fig. 6F and Fig. S6D). Collectively, these findings illustrate the potential therapeutic benefits of combined treatment of HCC with macropinocytosis inhibitors and sorafenib.

## Discussion

The findings reported herein are the first to demonstrate a role for macropinocytosis in evasion of sorafenib-induced ferroptosis by HCC; as such, they underscore the necessity of targeting macropinocytosis when treating advanced HCC with sorafenib. We found that macropinocytosis-driven catabolism of extracellular protein enables HCC cells to become resistant to sorafenib-induced ferroptosis by replenishing GSH through acquisition of cysteine. Accordingly, systemic inhibition of macropinocytosis renders HCC more susceptible to sorafenib-induced ferroptosis by blocking the alternative pathway of cysteine acquisition (Fig. 6G).

Homeostatic dysfunction of ferroptosis is believed to be an essential cause of chemotherapy resistance; therefore, several strategies, including inhibition of cystine uptake, have been tried to sensitize cancer cells to ferroptosis [33]. Multiple studies verified that sorafenib acts as a strong inducer of ferroptosis by inhibiting the activity of system xc- irrespective of oncogene and kinase status [23, 24]. Given that negative regulators of ferroptosis such as nuclear factor erythroid 2-related factor 2, metallothionein-1G, and p53 confer resistance to sorafenib in HCC [8, 34], the discovery of a novel mechanism underlying ferroptosis induction is a promising therapeutic avenue to enhancing the antitumor efficacy of sorafenib against HCC. Here, we confirmed that sorafenib induces ferroptosis in HCC, along with a marked decrease in intracellular cysteine levels. Interestingly, we found that supplementation with BSA not only rescues sorafenib-induced depletion of cysteine, but also prevents sorafenib-induced ferroptosis. The inhibitory effect of BSA on ferroptosis was abolished when macropinocytosis was inhibited, indicating that sorafenib-treated HCC cells have an alternative means of mitigating ferroptosis by scavenging extracellular proteins through macropinocytosis.

Originally, macropinocytosis was reported in cancer cells bearing KRAS or PTEN mutations [13, 15, 35]. Oncogenic mutations that activate the KRAS or PI3K pathways contribute to remodeling of the cytoskeleton, thereby inducing macropinocytosis. However, this study showed that, regardless of these oncogenic mutations, sorafenib induces macropinocytosis in human HCC specimens and xenografted HCC tissues, as well as in HCC cell lines. Supporting our findings, recent studies demonstrate that PI3K and its effector RAC1 activate macropinocytosis in response to growth factor stimulation, independent of RAS [16]. We also demonstrated that sorafenib-induced activation of PI3K drives macropinocytosis by activating RAC1-PAK1 signaling in HCC cells and HCC tissues in response to sorafenib treatment. In addition, KRAS-independent, but AMPK-dependent, macropinocytosis in cancer-associated fibroblasts is necessary for PDAC tumor growth [17]. Therefore, PI3K and AMPK are not only the primary effectors of responses to mitochondrial dysfunction [36, 37], but are also required for macropinocytosis [38]. These results, combined with our observations, suggest that sorafenib induces mitochondrial dysfunction in HCC, and that subsequent stimulation of PI3K and AMPK is responsible for induction of macropinocytosis, independent of oncogenic signals.

Prior to this report, studies showed that the main role of macropinocytosis was to scavenge nutrients as a fuel to drive cancer cell survival and proliferation [39]. Notably, we found that sorafenib-induced macropinocytosis has more dire consequences: it can enable scavenging of extracellular proteins to offset cysteine depletion, conferring resistance to ferroptotic cell death. In support of these findings, HCC patients with higher levels of RAC1, CDC42, and PAK1 show worse overall survival, which can be attributed to enhanced macropinocytosis and resistance to cancer therapy. Since macropinocytosis blockers are available in a clinical setting, the results presented herein suggest a clinical application for these compounds in HCC. EIPA, a NHE inhibitor, blocks macropinocytosis by lowering sub-membranous pH [40]. In addition, amiloride, a currently prescribed diuretic, blocks macropinocytosis by inhibiting RAC1 and CDC42 signaling [41]. The preset study showed that amiloride had a robust anti-tumor effect when macropinocytosis was induced by sorafenib but that amiloride alone did not; thus, inhibition of macropinocytosis by amiloride is responsible for the enhanced anti-tumor efficacy of sorafenib. Taken together, the data in the present study show that blocking macropinocytosis intensified sorafenib-induced HCC cell ferroptosis, thereby overcoming sorafenib resistance and enhancing the effects of sorafenib against treatment-naïve HCC and sorafenib-adapted HCC xenografts.

## Conclusion

The results presented herein identify a role for macropinocytosis in mitigating ferroptosis. Sorafenib is a driver of macropinocytosis induction, and we show here how macropinocytosis acts to instigate resistance to sorafenib treatment in HCC. Our discovery suggests that targeting macropinocytosis has the potential to limit development of sorafenib resistance and improve the therapeutic efficacy of sorafenib in HCC. Therefore, the strategy of adding a macropinocytosis inhibitor such as amiloride, a clinically feasible NHE inhibitor, to sorafenib may produce significant and clinically relevant gains in survival for patients with advanced HCC.

## Supplementary Information

**Additional file 1.**

## Data Availability

The data supporting the conclusions of this article are provided in this article and the additional files. In addition, all data from this study can be obtained from the corresponding author upon reasonable request.

## Abbreviations

HCC: Hepatocellular carcinoma
ROS: Reactive oxygen species
FBS: Fetal bovine serum
PBS: Phosphate-buffered saline
OCR: Oxygen consumption rate
GSEA: Gene set enrichment analysis
TMR: Tetramethylrhodamine
EIPA: 5-(N-ethyl-N-isopropyl)-amiloride
NHE: Na^+^/H^+^ exchanger
BODIPY: Boron-dipyrromethene
BSA: Bovine serum albumin
4-HNE: 4-Hydroxynonenal
GSH: Glutathione
β-ME: β-Mercaptoethanol
BSO: Buthionine sulfoxamine
TCGA: The Cancer Genome Atlas
