# Supplementary material for: Macropinocytosis is an alternative pathway of cysteine acquisition and mitigates sorafenib-induced ferroptosis in hepatocellular carcinoma
Source: J Exp Clin Cancer Res. 2022 Mar 14;41:98. doi: 10.1186/s13046-022-02296-3 (PMC8919615; doi:10.1186/s13046-022-02296-3)
Supplement: Supplementary file 1 — Additional file 1. [file 13046_2022_2296_MOESM1_ESM.docx]

**Macropinocytosis is an alternative pathway of cysteine acquisition and mitigates sorafenib-induced ferroptosis in hepatocellular carcinoma**

Jun-Kyu Byun, Seunghyeong Lee, Gil Won Kang, Yu Rim Lee, Soo Young Park, Im-Sook Song, Jae Won Yun, Jaebon Lee, Yeon-Kyung Choi, Keun-Gyu Park

**Supplementary methods**

**Cell culture and chemical treatment**

The human liver cancer cell lines Huh7 and SK-Hep1 were cultured in DMEM medium containing 10% fetal bovine serum (FBS) and 1% penicillin/streptomycin (P/S). Other human liver cancer cell lines (PLC/PRF/5 and Hep3B) were cultured in RPMI 1640 and EMEM medium, respectively, containing 10% FBS and 1% P/S. Human kidney cancer cell lines (Caki-1 and ACHN) and mouse liver cancer cell line RIL-175 were cultured in DMEM containing 10% FBS and 1% P/S. RIL-175 were a kind gift from Lars Zender (University Hospital Tübingen, Germany). To demonstrate the effects of sorafenib or erastin on macropinocytosis, cells were treated with 10 μM sorafenib (Cayman Chemical, Ann Arbor, MI, USA) or 2 μM erastin (Sigma, St. Louis, MO, USA) for 24 h. Pharmacological inhibitors of AMPK (10 μM; Compound C; Sigma), PI3K (50 μM; LY294002; Abcam, Cambridge, UK), RAC1 (100 μM; NSC23766; Sigma), PAK1 (20 μM; IPA-3; Sigma), and Na^+^/H^+^ exchangers (40 μM EIPA, 500 μM Amiloride; Sigma) were applied for 2 h after 22 h of treatment with sorafenib. To demonstrate the effects of oligomycin and antimycin A on macropinocytosis, cells were treated for 6 h with 2.5 μM oligomycin (Sigma) and 10 μM antimycin A (Sigma). To determine the effects of ferroptosis in sorafenib-treated cells, cells were treated with 100 μM Trolox (Abcam, Cambridge, UK), 1 μM ferrostatin-1 (Sigma), 100 μM deferoxamine (Sigma), 5 mM glutathione reduced-ethyl ester (Sigma), 20 μM Z-VAD-FMK (Promega, Madison, WI, USA), 20 μM necrostatin-1 (Sigma), 100 μM buthionine sulfoxamine (BSO) (Sigma), 10 nM bafilomycin A1 (Sigma), or 25 μM β-mercaptoethanol (Sigma).

**Visualization of differentially expressed genes and survival data**

Clinical data and gene expression data from The Cancer Genome Atlas (TCGA) liver HCC were downloaded from the GDAC and the UCSC Xena browser (https://xena.ucsc.edu/). Box plots were visualized in the ggplot2 package of R ([1](#_ENREF_1)) and the t test was performed to assess statistical significance. Kaplan-Meier plots were constructed using the survminer package in R (https://cran.r-project.org/web/packages/survminer/). For each target gene, 25% of patients showing the highest RNA expression (the high expression-group) and 25% of patients showing the lowest expression were selected.

**Measurement of the oxygen consumption rate (OCR)**

The OCR was measured in a XF24 Extracellular Flux Analyzer (Seahorse Bioscience, North Billerica, MA, USA). Vehicle, sorafenib (10 μM), oligomycin (1 μM), CCCP (5 μM), rotenone (1 μM), or antimycin A (5 μM) were added at the indicated time points during OCR measurement. The OCR was normalized to the cell number and expressed as a ratio relative to baseline.

**Western blot analysis**

Proteins from cellular lysates were resolved on NuPAGE 4%–12% (Thermo Fisher Scientific, Waltham, MA, USA) or Tris-Glycine gels and transferred to PVDF membranes. Membranes were probed with antibodies specific for phospho-AKT (T308), AKT, phosphor-AMPK (T172), AMPK, phospho-PAK1 (S199/204)/PAK2 (S192/197), PAK1, SLC7A11 (all from Cell Signaling Technology, Danvers, MA, USA), CBS (Santa Cruz Biotechnology, Santa Cruz, CA, USA), and β-actin (Sigma). Secondary antibody was Rabbit/Mouse IgG Antibody (GenTex, Irvine, CA, USA).

**Active RAC1 detection assay**

RAC1-GTP levels were measured using the Active Rac1 Pull-Down and Detection Kit (Thermo Fisher Scientific).

**RNA isolation**

SK-Hep1 cells were treated with sorafenib, either alone or in combination with BSA or/and EIPA, for 24 h. Total RNA was extracted using TRIzol reagent (Thermo Fisher Scientific) and RNA quality was confirmed on an Agilent 2100 bioanalyzer using the RNA 6000 Nano Chip (Agilent Technologies, Amstelveen, Netherlands). RNA quantification was performed on an ND-2000 Spectrophotometer (Thermo Fisher Scientific).

**Library preparation and sequencing**

The library was constructed using the QuantSeq 3’ mRNA-Seq Library Prep Kit (Lexogen, Wien, Austria). In brief, 500 ng of total RNA was used per sample. An oligo-dT primer containing an Illumina-compatible sequence at its 50 end was hybridized to the RNA and reverse transcription was performed. After degradation of the RNA template, second-strand synthesis was initiated by a random primer containing an Illumina-compatible linker sequence at its 5’ end. The double-stranded library was purified using magnetic beads to remove all reaction components. The library was amplified to add the complete adaptor sequences required for cluster generation. The finished library was purified from the PCR reagents. High-throughput sequencing was performed as single-end 75 ntd sequencing on a NextSeq 500 (Illumina, Foster City, CA, USA).

**Data analysis**

QuantSeq 30 mRNA-Seq reads were aligned using Bowtie2 ([2](#_ENREF_2)). Bowtie2 indices were generated from either the genome assembly sequence or representative transcript sequences for alignment to the genome and transcriptome. The alignment file was used to assemble transcripts, estimate their abundances, and detect differential expression of genes. Differentially expressed genes were identified based on counts from unique and multiple alignments using coverage in Bedtools ([3](#_ENREF_3)). Read count (RC) data were processed using the quantile normalization method in EdgeR within R ([4](#_ENREF_4)), and Bioconductor ([5](#_ENREF_5)).

**Gene set enrichment analysis (GSEA)**

GSEA was performed to determine whether expression of a previously defined sets of genes shows significant difference between vehicle-treated and sorafenib-treated SK-Hep1 cells ([6](#_ENREF_6)). For this analysis, PI3K, RAS, and WNT signaling were analyzed using a gene set from WikiPathways ([7](#_ENREF_7)). A gene set provided by Dixon et al was used as a ferroptosis expression signature ([8](#_ENREF_8)). Gene sets provided by Bebber et al were used as apoptosis and necroptosis signatures ([9](#_ENREF_9)).

**Detection of lipid ROS**

For fluorescence detection of lipid ROS, cells were stained for 30 minutes with C11-BODIPY (10 μM; Thermo Fisher Scientific), washed three times with PBS, and the nuclei were stained using NucBlue^TM^ Live ReadyProbes^TM^ Reagent (Thermo Fisher Scientific). Data were displayed as the ratio of the intensity of green/red fluorescence in cells.

**siRNA transfection**

Cells were transfected with control siRNA or human siCBS (Bioneer, Daejeon, Korea) using Lipofectamine RNAiMAX (Thermo Fisher Scientiﬁc).

**Quantitative RT-PCR**

Total RNA was harvested using TRIzol reagent, and cDNA was synthesized using the RevertAid First Strand cDNA Synthesis Kit (Thermo Fisher Scientiﬁc), SYBR green PCR Master Mix (Applied Biosystems, Foster City, CA, USA), and a VIIA 7 Real-time PCR system instrument (Applied Biosystems). The primer sequences were as follows: CBS forward, AACATGCTCTCGTCCCTGCTT, and reverse, GAGGCGGATCTGTTTGAACTG; CTH forward, TTTGGCTCTGGGAGCTGATATT, and reverse, CCAGGCCCATTACAACATCAC; MAT2A forward, TGGAGACCAGGGCTTAATGTTT, and reverse, GCATTTAGCTTGTGTGCCAAGA; GNMT forward, ATGCTGTCATCTGCCTTGGAA, and reverse, GTAGGCCCCCTTTGCAGTCT; AHCY forward, GGCTGGCATTCCGGTGTAT, and reverse, GGCCCGTCCTTGAAGTACAG; MTR forward, GGCCTACCGGATGAACATGT, and reverse, GTTACCTCCTCGGCAGCTTTT; 36B4 forward, CCACGCTGCTGAACATGCT, and reverse, TCGAACACCTGCTGGATGAC. Gene expression was normalized to that of the endogenous reference gene, 36B4.

**Cell counting and clonogenic assay**

Cells were treated for 48 h with sorafenib, either alone or in combination with 3% bovine serum albumin (BSA; Sigma), EIPA, or/and amiloride. Cell counting was conducted by trypan blue staining using a hemocytometer. For clonogenic assay, cells were treated with chemicals for 7 days. The medium was replaced every 2 days. Cells were fixed and stained with 0.5% crystal violet.

**Measurement of cysteine concentrations**

Cells were treated for 24 h with sorafenib, either alone or in combination with 3% BSA, EIPA, and/or bafilomycin A1. The intracellular cysteine concentration in cells was measured using a cysteine assay kit (Biovision, Milpitas, CA, USA). Data were normalized against cell number.

**Measurement of GSH concentration**

Cells were treated for 24 h with sorafenib, either alone or in combination with 3% BSA and/or EIPA. Intracellular GSH concentration in cells was measured using the GSH-Glo Glutathione assay (Promega). Data were normalized against cell number.

**Generation of sorafenib-resistant HCC cells**

HCC cells were treated with sorafenib for several weeks until sorafenib-treated cell numbers reach more than 80% compared to control. Over a period of 5 months, cells were exposed to continuous administration of sorafenib at gradually increasing concentrations.

**Hematoxylin and eosin staining**

Tumor tissues were extracted at the experimental endpoint, fixed with 4% paraformaldehyde (Biosesang, Seongnam, Korea), and embedded in paraffin. Hematoxylin and eosin staining of paraffin-embedded sections were carried out using standard procedures.

**Animal experiments**

Cancer cells (6 × 10^6^) were injected subcutaneously into 7-week-old male nude mice. After confirming a palpable xenografted tumor, mice received a daily intraperitoneal (i.p.) injection of vehicle, sorafenib (5 mg/kg), or/and amiloride (5 mg/kg) until the experimental end point. Tumor volume was calculated as length × width^2^ × 0.5 (mm^3^), and body weight was measured every 2 days. For intrahepatic inoculation of mouse liver cancer cells, 1 × 10^5^ RIL-175 cells were injected into the left lobe of in C57BL/6 mouse livers (6-week-old male). Drugs were injected as described above. The last dose of drug was administered 4 h before the experimental endpoint. All animal procedures were approved by the Institutional Animal Care and Use Committee of Kyungpook National University.

**Statistical analysis**

Statistical analyses were performed using Student’s t test. Data in graphs are presented as the mean ± SEM. The open software R (version 3.4.3) was used for all statistical analyses and data processing. The heatmap was generated using the ComplexHeatmap R package ([10](#_ENREF_10)). P values < 0.05 were considered statistically significant.

**
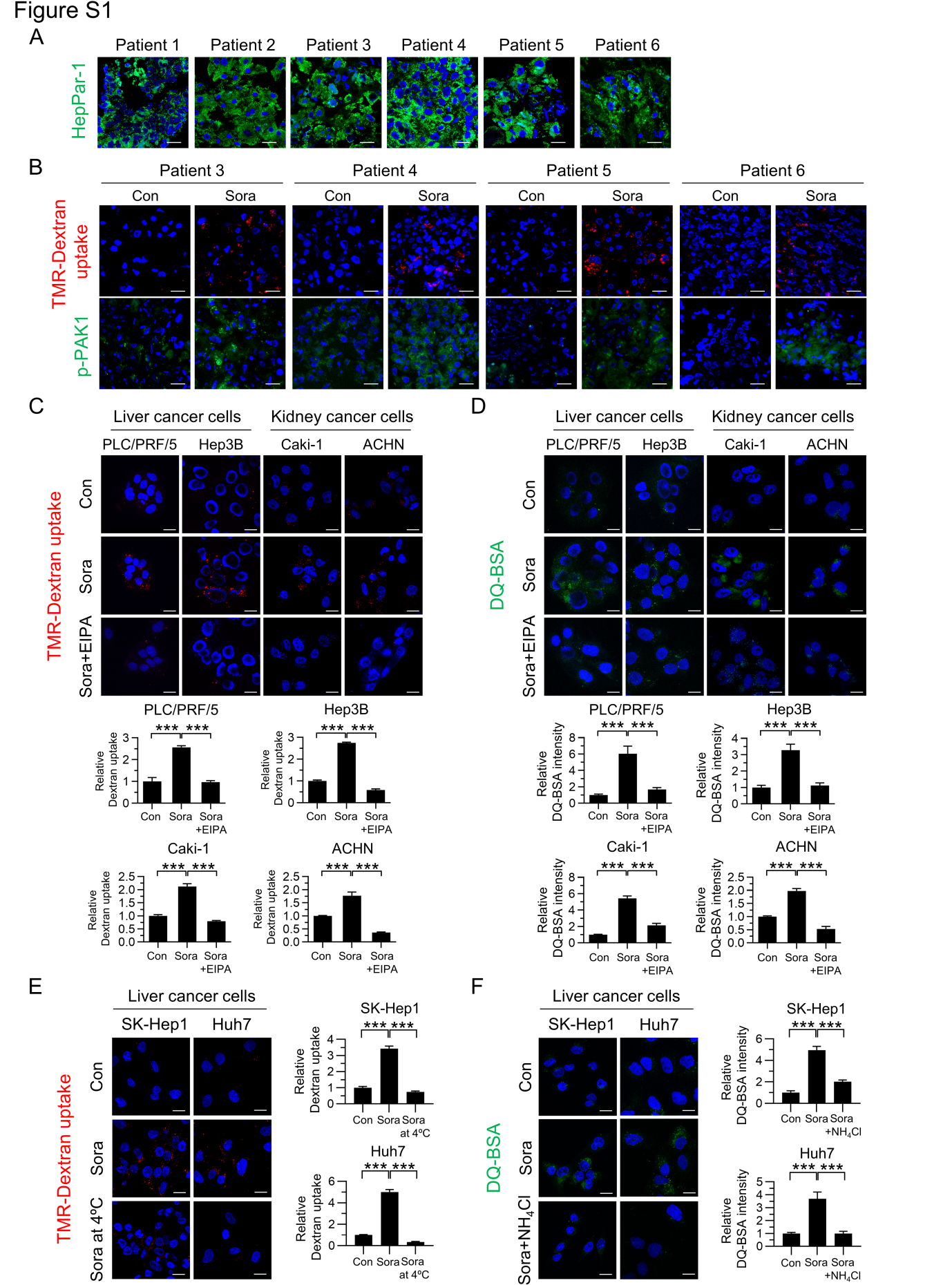
**

**Supplementary Figure S1. Sorafenib-treated HCC cells take up and degrade extracellular proteins through macropinocytosis.** (A) Representative images showing immunofluorescence staining of primary human HCC tissue for HepPar-1 (green). (B) Macropinocytotic uptake of labeled TMR-dextran (red) and immunofluorescence staining of phosphorylated PAK1 (green) in tumor tissues from treatment-naïve HCC patients exposed to sorafenib. (C) Representative images showing macropinosomes (red) in sorafenib-treated PLC/PRF/5, Hep3B, Caki-1, and ACHN cells in the presence or absence of EIPA (upper panel), and quantification of macropinosomes (lower panel). (D) Representative images showing DQ-BSA fluorescence (green) in sorafenib-treated PLC/PRF/5, Hep3B, Caki-1, and ACHN cells in the presence or absence of EIPA (upper panel), and quantification of DQ-BSA fluorescence (lower panel). (E) Representative images of macropinosomes (red) in sorafenib-treated SK-Hep1 and Huh7 cells incubated at 37°C or 4°C (left panel), followed by quantification of macropinosomes (right panel). (F) Representative images of DQ-BSA fluorescence (green) in sorafenib-treated SK-Hep1 and Huh7 cells in the presence or absence of NH_4_Cl (left panel), and quantification of fluorescence (right panel). Data are normalized against values measured in vehicle-treated cells (Con) and expressed as the mean ± SEM of at least three independent experiments. Scale bar, 20 µm. *** p<0.001.

**
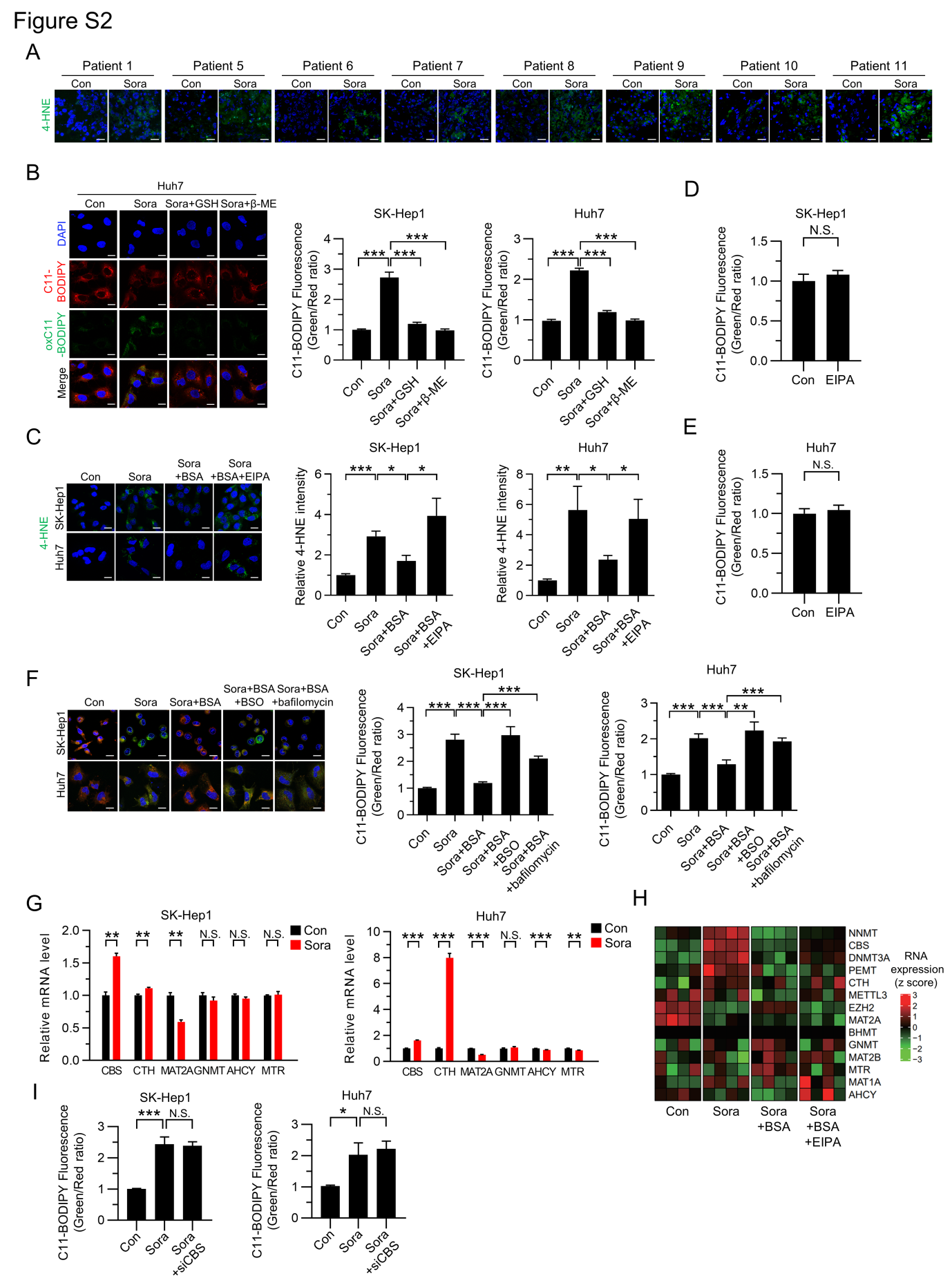
**

**Supplementary Figure S2**. **Macropinocytosis prevents sorafenib-induced ferroptosis.** (A) Immunofluorescence staining of 4-HNE (green) in response to sorafenib in tumor tissues from treatment-naïve HCC patients. (B) Representative images showing C11-BODIPY, a marker of lipid peroxidation, in Huh7 cells treated with sorafenib, either alone or in combination with the antioxidant GSH or the reducing agent β-mercaptoethanol (β-ME) (left panel). C11-BOIPY indicates the levels of staining by the probe (unoxidized), while oxC11-BODIPY (oxidized) indicates the level of lipid ROS. Quantification of C11-BODIPY fluorescence in SK-Hep1 and Huh7 cells (right panel). (C) Immunofluorescence staining with anti-4-HNE (green) in SK-Hep1 and Huh7 cells treated with sorafenib, either alone or in combination with BSA and/or EIPA (left panel). Quantification of 4-HNE fluorescence (right panel). (D and E) Quantification of C11-BODIPY fluorescence in SK-Hep1 (D) and Huh7 cells (E) treated with EIPA. (F) Representative images showing C11-BODIPY in SK-Hep1 and Huh7 cells treated with sorafenib, either alone or in combination with BSA, buthionine sulfoxamine (BSO), and/or bafilomycin A1 (left panel). Quantification of C11-BODIPY fluorescence in the cells (right panel). (G) Relative expression of mRNA encoding the indicated genes in sorafenib-treated SK-Hep1 and Huh7 cells. (H) Genes shown in the heatmap of SK-Hep1 cells treated with sorafenib, either alone or in combination with BSA or/and EIPA. (I) Quantification of C11-BODIPY fluorescence in the cells shown in Fig. 2I. Data are normalized against values measured in vehicle-treated cells (Con) and expressed as the mean ± SEM of at least three independent experiments. Scale bar, 20 µm. N.S., not significant; *p<0.05, **p<0.01, and ***p < 0.001.

**
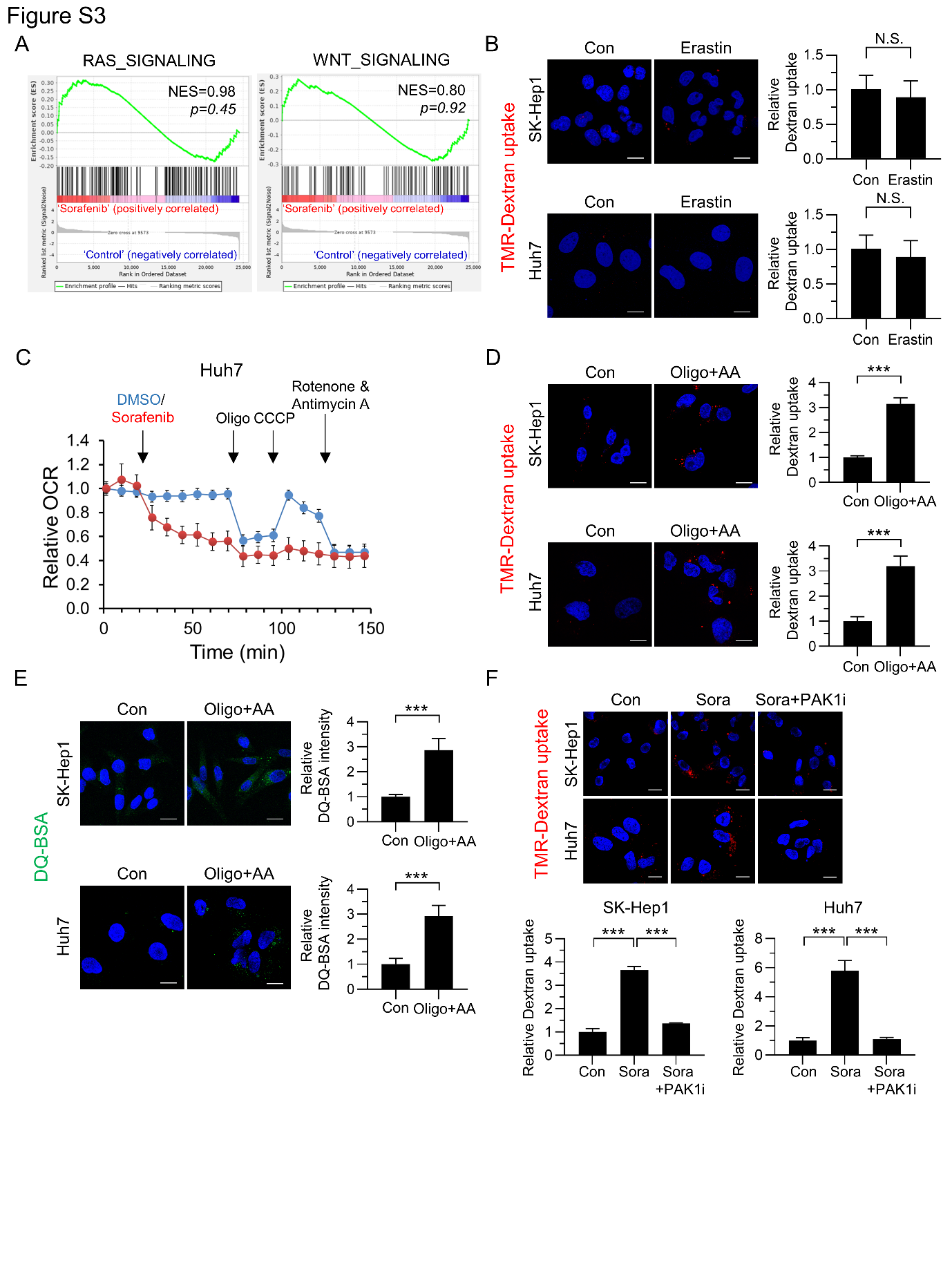
**

**Supplementary Figure S3. Sorafenib-induced mitochondrial dysfunction induces macropinocytosis.** (A) Gene set enrichment analysis of RAS or WNT signaling among genes expressed by sorafenib-treated SK-Hep1 cells. (B) Representative images of macropinosomes (red) in SK-Hep1 and Huh7 cells treated with erastin (left panel). Quantification of macropinosomes (right panel). (C) The oxygen consumption rate (OCR) of Huh7 cells at the indicated time points after sorafenib treatment. (D) Representative images of macropinosomes (red) in SK-Hep1 and Huh7 cells treated with oligomycin (Oligo) and antimycin A (AA) (left panel). Quantification of macropinosomes (right panel). (E) Representative images of DQ-BSA fluorescence (green) in SK-Hep1 and Huh7 cells treated with oligomycin and antimycin A (left panel). Quantification of DQ-BSA fluorescence (right panel). (F) Representative images showing macropinosomes (red) in sorafenib-treated SK-Hep1 and Huh7 cells in the presence or absence of a PAK1 inhibitor (IPA-3) (upper panel). Quantification of macropinosomes in the cells (lower panel). Data are normalized against values measured in vehicle-treated cells (Con) and expressed as the mean ± SEM of at least three independent experiments. Scale bar, 20 µm. N.S., not significant and ***p<0.001.

**
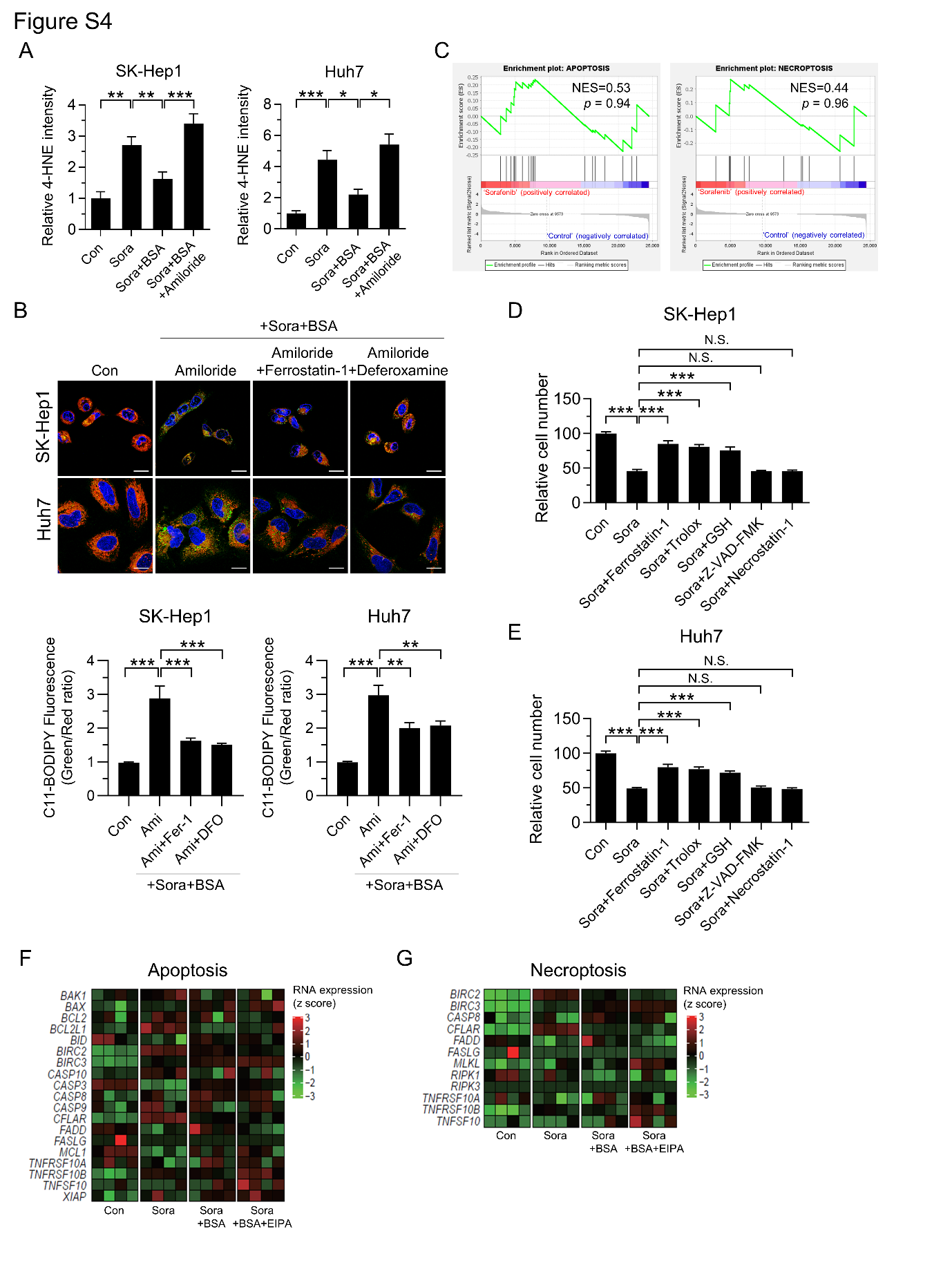
**

**Supplementary Figure S4. Effects of sorafenib on ferroptosis, apoptosis, and necroptosis of HCC cells.** (A) Quantification of 4-HNE fluorescence in cells shown in Fig. 4D. (B) Representative images of C11-BODIPY staining in SK-Hep1 and Huh7 cells treated with sorafenib, either alone or in combination with BSA and/or amiloride (Ami), ferrostatin-1 (Fer-1), or deferoxamine (DFO) (upper panel). Quantification of C11-BODIPY fluorescence in cells (lower panel). (C) Gene set enrichment analysis of apoptosis- and necroptosis-related genes expressed by sorafenib-treated SK-Hep1 cells. (D and E) Relative numbers of SK-Hep1 (D) and Huh7 (E) cells after treatment for 24 h with sorafenib, either alone or in combination with ferrostatin-1, trolox, GSH, Z-VAD-FMK, or necrostatin-1. (F) Apoptosis- and (G) necroptosis-related genes shown in the heatmap of SK-Hep1 cells treated with sorafenib, either alone or in combination with BSA and/or EIPA. Data are normalized against values measured in vehicle-treated cells (Con) and expressed as the mean ± SEM of three independent experiments. N.S., not significant; *p<0.05, **p<0.01, and ***p < 0.001

**
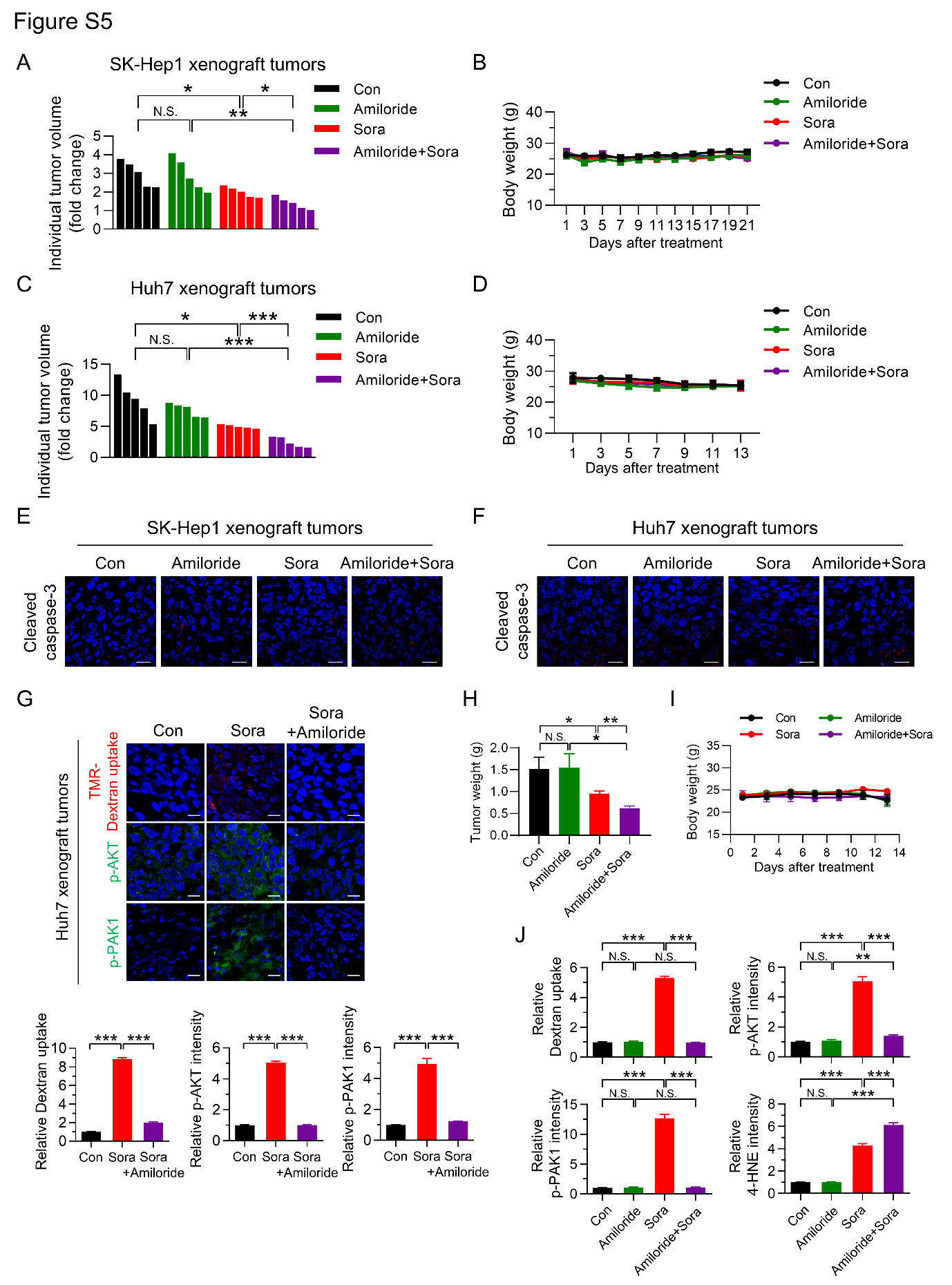
**

**Supplementary Figure S5. Effects of co-treatment with sorafenib and amiloride on tumor growth and body weight of HCC-xenografted mice.** (A) Tumor volume at the experimental endpoint and (B) body weight of mice harboring SK-Hep1 tumor xenografts treated with drugs (see Fig. 5A). (C) Tumor volume at the experimental endpoint

and (D) body weight of mice harboring Huh7 xenografts treated with drugs (see Fig. 5B). Body weight is presented as the mean ± SEM (n = 5 per group). (E and F) Immunofluorescence staining of SK-Hep1 (E) and Huh7 (F) xenograft tissue with anti-cleaved caspase-3. Scale bar, 20 µm. (G) Representative images of macropinosomes (red) and immunofluorescence staining for anti-phosphorylated AKT and PAK1 (green) in sections of Huh7 xenografted tumor tissue (upper panel). Quantification of macropinosomes and phosphorylated AKT, and PAK1 fluorescence in tumor tissues (lower panel) (n = 3 per group). (H and I) Tumor weight (H) and body weight (I) of C57BL6 mice implanted with RIL-175 cells shown in Fig. 5F. (J) Quantification of macropinosomes, phosphorylated AKT and PAK1, and 4-HNE staining in tumor tissues shown in Fig. 5G (n = 6–8 per group). N.S., not significant; *p<0.05, ** p <0.01, and *** p<0.001.

**
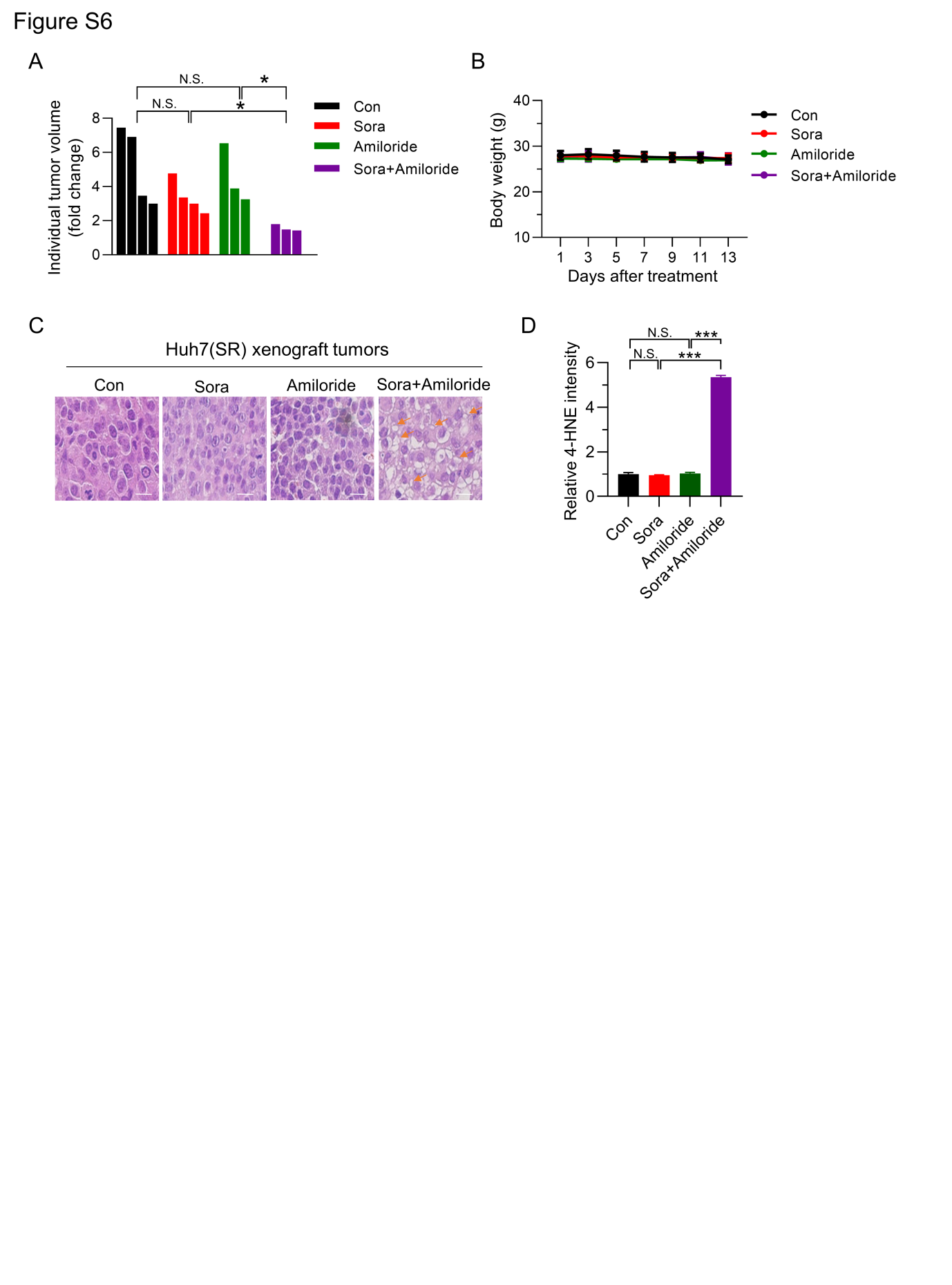
**

**Supplementary Figure S6. Effects of sorafenib plus amiloride on tumor growth and body weight of mice bearing sorafenib-resistant HCC xenografts.** (A) Tumor volume at the experimental endpoint and (B) body weight of mice bearing Huh7 (SR) xenografts treated with drugs (see Fig. 6E). Body weight is expressed as the mean ± SEM (n = 3–4 per group). (C) H&E staining of tumor tissues in Huh7 (SR) xenografts. Arrows indicate lipid droplet formation. (D) Quantification of immunofluorescence tissue staining with 4-HNE (See Fig. 6F). Scale bar, 20 µm. N.S., not significant; *p<0.05 and ***p<0.001.

**Supplementary Table S1. Clinical characteristics of patients with hepatocellular carcinoma who underwent ultrasound-guided needle biopsy**

| Variable | No. of patients |
| --- | --- |
| All cases | 11 |
| Gender (male/female) | 10/1 |
| Mean age (years) | 68.82 ± 11.39 |
| Cause of hepatocellular carcinoma |  |
| Alcohol | 5 |
| Hepatitis B virus | 3 |
| Hepatitis B virus + Alcohol | 1 |
| Other | 2 |
| Tumor size |  |
| ≤ 2 cm | 2 |
| > 2 to ≤ 3 cm | 1 |
| > 3 to ≤ 5 cm | 3 |
| > 5 cm | 5 |

**References**

1. Wickham H. ggplot2: Elegant Graphics for Data Analysis: Springer-Verlag New York; 2016.

2. Langmead B, Salzberg SL. Fast gapped-read alignment with Bowtie 2. Nat Methods. 2012;9(4):357-9.

3. Quinlan AR, Hall IM. BEDTools: a flexible suite of utilities for comparing genomic features. Bioinformatics. 2010;26(6):841-2.

4. Team RC. R: A Language and Environment for Statistical Computing. R Foundation for Statistical Computing; 2016.

5. Gentleman RC, Carey VJ, Bates DM, Bolstad B, Dettling M, Dudoit S, et al. Bioconductor: open software development for computational biology and bioinformatics. Genome Biol. 2004;5(10):R80.

6. Subramanian A, Tamayo P, Mootha VK, Mukherjee S, Ebert BL, Gillette MA, et al. Gene set enrichment analysis: a knowledge-based approach for interpreting genome-wide expression profiles. Proc Natl Acad Sci U S A. 2005;102(43):15545-50.

7. Pico AR, Kelder T, van Iersel MP, Hanspers K, Conklin BR, Evelo C. WikiPathways: pathway editing for the people. PLoS Biol. 2008;6(7):e184.

8. Dixon SJ, Patel DN, Welsch M, Skouta R, Lee ED, Hayano M, et al. Pharmacological inhibition of cystine-glutamate exchange induces endoplasmic reticulum stress and ferroptosis. Elife. 2014;3:e02523.

9. Bebber CM, Thomas ES. Ferroptosis response segregates small cell lung cancer (SCLC) neuroendocrine subtypes. Nat Commun. 2021;12(1):2048.

10. Gu Z, Eils R, Schlesner M. Complex heatmaps reveal patterns and correlations in multidimensional genomic data. Bioinformatics. 2016;32(18):2847-9.
